# Exosomal TRIM3 is a novel marker and therapy target for gastric cancer

**DOI:** 10.1186/s13046-018-0825-0

**Published:** 2018-07-21

**Authors:** Hailong Fu, Huan Yang, Xu Zhang, Bo Wang, Jiahui Mao, Xia Li, Mei Wang, Bin Zhang, Zixuan Sun, Hui Qian, Wenrong Xu

**Affiliations:** 1 Jiangsu Key Laboratory of Medical Science and Laboratory Medicine, School of Medicine, Jiangsu University, 301 Xuefu Road, Zhenjiang, 212013 Jiangsu China; 2 Center for Clinical Laboratory, the First Affiliated Hospital of Soochow University, 188 Shizi Street, Suzhou, 215006 Jiangsu China; 3 Department of Clinical Laboratory, The Second Affiliated Hospital of Soochow University, 1055 Sanxiang Road, Suzhou, 215004 Jiangsu China; 4 Department of Oncology, The Second Affiliated Hospital of Soochow University, 1055 Sanxiang Road, Suzhou, 215004 Jiangsu China

**Keywords:** Gastric cancer, Exosomes, TRIM3, Progression, Diagnosis, Therapy

## Abstract

**Background:**

Exosomes are critically involved in cancer development and progression. The exosomal contents have been suggested as ideal cancer biomarkers. In this study, we investigated the expression of exosomal proteins in the serum of gastric cancer patients and their roles in gastric cancer.

**Methods:**

The proteomic profile of exosomes from the serum of gastric cancer patients was detected by using LC-MS/MS. The expression of TRIM3 in exosomes from the serum of gastric cancer patients and healthy controls was assessed by ELISA and western blot. Immunohistochemistry was used to detect TRIM3 expression in gastric cancer tissues and their matching adjacent tissues. The growth and migration abilities of gastric cancer cells with TRIM3 overexpression or knockdown in vitro were evaluated by colony formation assay and transwell migration assay. The effects of TRIM3 overexpression or knockdown on gastric cancer growth and metastasis in vivo were investigated by using subcutaneous xenograft tumor and peritoneal metastasis mouse model. The effects of TRIM3-overexpressing exosomes on gastric cancer growth and metastasis in vitro and in vivo were also evaluated.

**Results:**

We found that the expression levels of TRIM3 mRNA and protein were decreased in gastric cancer tissues compared to the matched control tissues. In addition, the levels of TRIM3 protein in the serum exosomes of gastric cancer patients were lower than that in healthy controls. We demonstrated that TRIM3 overexpression reduced while TRIM3 knockdown promoted the growth and metastasis of gastric cancer in vitro and in vivo through the regulation of stem cell factors and EMT regulators. Moreover, exosomes-mediated delivery of TRIM3 protein could suppress gastric cancer growth and metastasis in vitro and in vivo.

**Conclusions:**

Taken together, our findings suggest that exosomal TRIM3 may serve as a biomarker for gastric cancer diagnosis and the delivery of TRIM3 by exosomes may provide a new avenue for gastric cancer therapy.

**Electronic supplementary material:**

The online version of this article (10.1186/s13046-018-0825-0) contains supplementary material, which is available to authorized users.

## Background

Gastric cancer (GC) is the fourth most common cancer in the world and the second leading cause of cancer-related deaths. It is estimated that there are approximately 750,000 new cases diagnosed annually around the world, and 5-year overall survival rates are less than 25% [1]. In spite of the progress in chemotherapy, radiotherapy and surgical techniques for GC in recent years, the survival rate of GC patients remains unsatisfactory [2]. One of the main reasons for the low overall survival is the lack of appropriate molecular biomarkers, which results in most of GC patients being diagnosed at advanced stage, missing the best opportunity for curative surgery.

Exosomes are small vesicles with several unique properties such as classic dish or cup morphology, 50–100 nm in diameter, double lipid layer, and a density of 1.12–1.19 g/mL [3, 4]. Exosomes are generated from the internal vesicles of multivesicular bodies (MVBs), which release their contents into biological fluids [5, 6]. Exosomes in the body fluids such as blood and urine could serve as diagnostic markers for various diseases including cancer because they may reflect the pathological state of their derived cells [7–9]. Exosomes contain proteins, nucleic acids, and lipids, which usually vary with the cellular and tissue origins of the exosomes and are adapted to their functions [10, 11]. The identification and isolation of cancer cells derived exosomes in body fluids could enable the specific detection of DNA, RNA, and proteins, which will aid in the diagnosis and treatment of cancer.

Tripartite motif (TRIM) proteins, one of the subfamilies of the RING-type E3 ubiquitin ligases, are regarded as critical regulators of neoplastic processes [12, 13]. These proteins play important roles in a variety of biological processes, such as cell proliferation, cell differentiation, DNA repair, transcriptional regulation, and apoptosis [12, 13]. Tripartite motif-containing 3 (TRIM3) is a member of the TRIM protein family, which maps to chromosome 11p15.5 [14, 15]. It has been reported that the loss of TRIM3 gene promotes the development and progression of glioblastomas, while overexpression reduces the tumorigenicity of GBM. The tumor suppressive function of TRIM3 in GBM may be linked to its regulation of p21 [16, 17]. TRIM3 has also been reported to be lowly expressed in glioma cells [18]. However, the roles of TRIM3 in gastric cancer have not been well characterized.

In the present study, we isolated exosomes from the serum of gastric cancer patients and identified the proteomic profile of serum exosomes. We found that TRIM3 protein level was decreased in the serum exosomes of gastric cancer patients compared to that in healthy controls. TRIM3 exerted a tumor suppressive role in gastric cancer. TRIM3 overexpression inhibited while TRIM3 knockdown promotedthe growth and metastasis of gastric cancer through the regulation of stem cell factors and EMT regulators. Moreover, we demonstrated that exosomes-mediated delivery of overexpressed TRIM3 could suppress gastric cancer growth and metastasis in vitro and in vivo.

## Methods

### Cell lines and cell culture

Human gastric cancer cell lines SGC-7901 and MGC-803, normal gastric epithelial cell line GES-1, and HEK-293 cell line were purchased from the Institute of Biochemistry and Cell Biology at the Chinese Academy of Sciences (Shanghai, China). The cells were propagated in RPMI-1640 medium (Gibco, Grand Island, USA) supplemented with 10% fetal bovine serum (FBS; Gibco). The cells were cultured at 37 °C in humidified air with 5% CO_2_.

### Human gastric cancer samples

Peripheral blood samples were collected after obtaining informed consent from 80 gastric cancer patients (63 male and 17 female), and 80 healthy volunteers (50 male and 30 female) used as age and sex matched control groups, which were collected from the Affiliated Peoples’ Hospital of Jiangsu University, China. Blood samples were centrifuged and serum was isolated and kept at − 80 °C until analysis. The serum samples of 20 gastric cancer patients (14 male and 6 female) and age matched 20 healthy volunteers (13 male and 7 female) were collected from the Affiliated Peoples’ Hospital of Jiangsu University. Paraffin sections of 20 (13 male and 7 female) gastric cancer and adjacent noncancerous tissues were collected from the Affiliated Peoples’ Hospital of Jiangsu University, China. The primary gastric cancer tissues and their matched, adjacent noncancerous tissues (located more than 5 cm away from the primary site) were collected from 60 gastric cancer patients (46 male and 14 female) undergoing surgery at the Affiliated Peoples’ Hospital of Jiangsu University, China. Informed consent was given in all patients examined. All samples were confirmed by pathological examination. Histological grade was defined according to the World Health Organization classification. Documented informed consent was obtained from all subjects and the Ethics Committee of Jiangsu University approved all aspects of the study (2012258).

### Exosome isolation

Exosomes were obtained from the serum and the culture supernatants by using ExoQuick precipitation solution (System Biosciences, Mountain View, CA, USA) according to manufacturer’s instructions. Briefly, 250 μL of serum were mixed with 63 μL of ExoQuick solution and incubated overnight at 4 °C. After centrifugation at 1500 *g* for 30 min, the pellets were suspended in 50 μL PBS. The culture supernatants from cells that had grown to sub-confluence (70–80%) in serum-free medium were harvested. The appropriate volume of ExoQuick-TC Exosome Precipitation Solution was added into the cell supernatant (1:5 ratio) and incubated at 4 °C overnight. After centrifugation at 1500 *g* for 30 min, the pellets were resuspended in PBS and filtrated through a 0.22 μm filter (EMD Millipore, Billerica, MA, USA). The isolated exosomes were stored at − 70 °C until use.

### Transmission electron microscopy

Twenty microliters of the prepared exosomes were pipetted onto formvar carbon-coated copper grids and allowed to adsorb for 10 min before excess fluid was drained. The adsorbed exosomes were then negatively stained with 2% (*w*/*v*) phospphotungstic acid (pH 6.8) for 5 min and was air-dried under an electric incandescent lamp, and analyzed with a transmission electron microscope (FEI Tecnai 12, Philips), bar = 50 nm.

### Size and concentration analyses of exosomes

The isolated exosomes were diluted in PBS and were analyzed using a NanoSight LM 10-HSBFT 14 Instrument (NanoSight, Malvern, UK) according to the manufacturer’s protocol. The size and concentration of exosomes were then analyzed by using the Nanoparticle Tracking Analysis 2.0 (NTA 2.0) software.

### LC-MS/MS analysis

Exosomes were obtained from the serum of gastric cancer patients and healthy controls (*n* = 3) with ExoQuick precipitation solution (System Biosciences, Mountain View, CA, USA) according to manufacturer’s instructions. The serum exosomes were re-suspended in 50 μl of PBS, 2 μl triton X-100, and 5 μl phenylmethylsulfonyl fluoride with vortexing to dissolve the vesicles. The insoluble fraction was pelleted by centrifugation 20,000 g. The insoluble fraction was acetone precipitated at − 20 °C and digested in-gel with 1μg/ul trypsin (sequencing grade, Promega) for 18 h at 37 °C. Resulting peptides were analyzed by LC-MS/MS on an Q-Exactive-Orbitrap mass spectrometer (Thermo Scientific, Waltham MA, USA). Fold change means the ratio of direct reporter group strength, this experiment selected 1.13 times as the difference threshold, and *P* value< 0.05.

### ELISA for TRIM3 detection

Exosomes were homogenized and lysed in RIPA buffer supplemented with proteinase inhibitors. TRIM3 concentration in exosomes was measured by using a commercial ELISA Kit according to the manufacturer’s instruction (Sanco, Hong Kong, China). The absolute amount of TRIM3 protein was calculated based on standard curves using human recombinant TRIM3 as the standard material. The concentration of TRIM3 was expressed as pictograms per milliliter.

### TRIM3 plasmid transfection

The TRIM3 expression vector and control vector were purchased from Genechem (Shanghai, China). The TRIM3 expression vector or control vector were transfected into MGC-803 and SGC-7901 cells by using Lipofectamine 2000 (Invitrogen, Carlsbad, CA, USA) according to the manufacturer’s instructions. The expression of TRIM3 was confirmed by using real-time quantitative RT-PCR and western blot at 48 h after transfection.

### siRNA transfection

Chemically synthesized TRIM3 siRNA and the scramble control siRNA were purchased from Genepharma (Shanghai, China). The sequences of siRNAs are shown in Additional file 1: Table S1. The siRNAs were transiently transfected into MGC-803 and SGC-7901 cells by using Lipofectamine 2000 reagent (Invitrogen, Carlsbad, CA, USA) according to the manufacturer’s instructions. Cells were plated in 6-well plates at a density of 1 × 10^5^ cells/well.

### Exosome treatment

MGC-803 and SGC-7901 cells were treated with various doses of control and TRIM3-overexpressing exosome (10 μg, 25 μg, 50 μg) and cultured for 48 h, MGC-803 cells were treated with TRIM3-overexpressing exosome derived from MGC-803 cells and SGC-7901 cells were treated with TRIM3-overexpressing exosome derived from SGC-7901 cells.

### Colony formation assay

Cells were harvested and seeded into 35 mm plates (1000 cells/well) and incubated for 10 days under standard conditions. At the end of the incubation period, the colonies were fixed with 4% paraformaldehyde and stained with crystal violet.

### Transwell migration assay

Cells (1 × 10^5^/well) were plated into the top chamber and 10% FBS containing medium was placed into the bottom chamber. After incubation at 37 °C in 5% CO_2_ for 12 h, the cells remaining at the upper surface of the membrane were removed with a cotton swab. The cells that migrated through the 8 μm sized pores and adhered to the lower surface of the membrane were fixed with 4% paraformaldehyde, stained with crystal violet and photographed.

### RNA extraction, RT-PCR and real-time RT-PCR

Total RNA was extracted from cells and tissues using TRIZOL Reagent (Invitrogen, Carlsbad, CA, USA) according to the manufacturer’s instructions. Total RNA was extracted from serum exosomes using QIAGEN RNA extraction Kit and equal amount of RNA was used for RT-PCR and real-time RT-PCR analyses. β-actin was used as the internal control. The sequences of specific primers are listed in Additional file 2: Table S2.

### Western blot

The cells and isolated exosomes were homogenized and lysed in RIPA buffer supplemented with proteinase inhibitors. Equal amount of proteins was loaded and separated on a 10% SDS-PAGE gel. Following electrophoresis, the proteins were transferred to a PVDF (polyvinylidene difluoride) membrane, blocked in 5% (*w*/*v*) non-fat milk and incubated with the primary antibodies. The sources of primary antibodies were: GAPDH and E-cadherin (Cell Signaling Technology, Beverly, MA, USA); N-cadherin (Abcam, USA); Sox2 (Millipore, USA); PCNA (Bioworld Technology, Louis Park, MN, USA); Oct4,Vimentin (Signalway Antibody, USA); GAPDH, Goat anti rabbit IgG (HRP), and Goat anti mouse IgG (HRP) (CWBIO, China).

### Animal model

The animal studies were performed with approval of the University Committee on Use and Care of Animals of Jiangsu University. The male BALB/c nu/nu mice aged 4–6 weeks were purchased from the Laboratory Animal Center of Shanghai (Academy of Science, Shanghai, China) and were randomly divided into six groups (*n* = 6).

### Tumor growth assay in vivo

Two groups received subcutaneous injections of either vector or TRIM3-overexpressing MGC-803 cells (2 × 10^6^ cells in 200 μl PBS), and two groups received subcutaneous injections of either NC-siRNA or TRIM3-siRNA transfected MGC-803 cells (2 × 10^6^ cells in 200 μl PBS). MGC-803 pre-treated with TRIM3-overexpressing or control exosomes were subcutaneously injected into the two-flank of BALB/c nude mice (2 × 10^6^ cells in 200 μl PBS). Tumors were surgically removed 20 days after injection, photographed and weighted. Tumor volume was assessed by caliper measurement and calculated by the formula (L × W × W/2), where L represents length, and W represents width.

### Tumor metastasis assay in vivo

The mice were grouped as described above. MGC-803 cells were intraperitoneally injected into the mice (2 × 10^6^ cells in 500 μl PBS). Mice were inspected every 2 days and killed at 1 month after injection. The metastatic tumor nodes were removed and examined.

### Vector construction and luciferase activity assay

The 3′ untranslated regions (3’-UTR) of TRIM3 mRNA were obtained by PCR and ligated into the pmirGLO dual-luciferase miRNA target expression vector (Promega, Madison, WI, USA). The mutant constructs were generated using Quick Change II Site-Directed Mutagenesis Kit (Agilent Technologies, Santa Clara, CA, USA). MiRNA-20a mimics and NC-mimics, miRNA-20a inhibitors and NC-inhibitor were purchased from Genepharma (Shanghai, China), and the sequences are shown in Additional file 3: Table S3. For the overexpression studies, HEK-293 cells were co-transfected with 500 ng reporter vector with miRNA-20a mimics or NC-mimics. For the knockdown studies, HEK-393 cells were co-transfected with 500 ng reporter vector with miRNA-20a inhibitors or NC-inhibitor. Firefly luciferase activity was examined after transfection by using a dual-luciferase reporter assay system (Promega, USA) and normalized to that of Renilla luciferase.

### Immunohistochemistry

The protein levels of TRIM3 in formalin-fixed paraffin-embedded gastric cancer tissues and adjacent non tumor tissue were detected by immunohistochemistry. Briefly, the sections were incubated with primary antibody and secondary antibody, visualized with 3,3′-diaminobenzidine (DAB) and then counterstained with hematoxylin for examination by the microscope. Twenty gastric cancer tissues and the corresponding adjacent noncancerous normal tissues were chosen randomly. The sections were photographed under a microscope and at least six fields were examined.

### Statistical analyses

All results were confirmed in at least three independent experiments, and data from one representative experiment were shown. Data are presented as the mean ± SD. A two-tailed Student’s t-test was used to test the differences in sample means for data with normally distributed means. Mann-Whitney U-test was used for non-parametric data. The statistical analysis was performed using SPSS Statistics software. Values of *P* < 0.05 were considered significant.

## Results

### The characterization of exosomes from the serum of gastric cancer patients

**Fig. 1:**
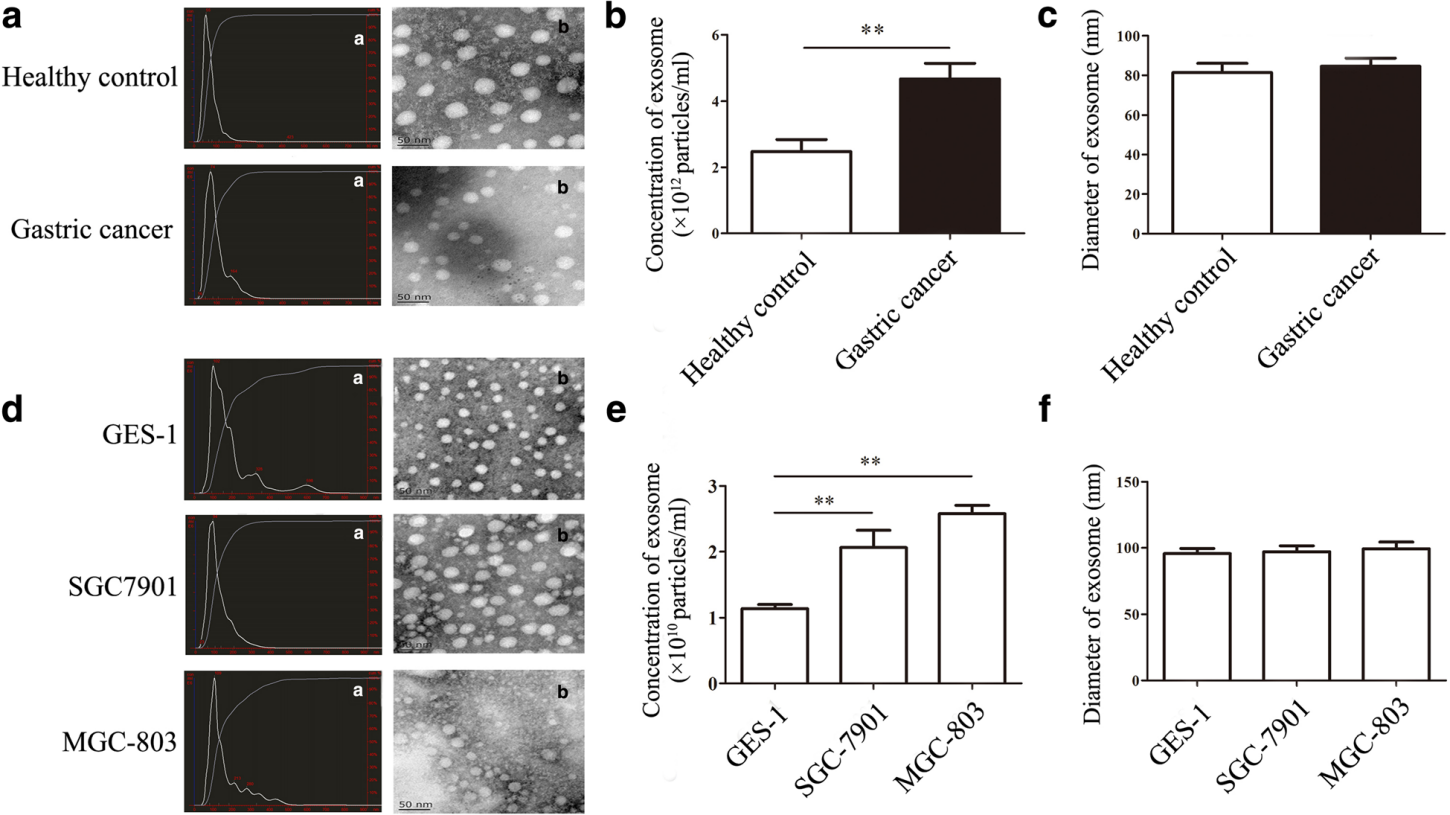
The characterization of exosomes from the serum of gastric cancer patients and the culture supernatant of gastric cancer cell lines. **a**, The size and morphology of exosomes from the serum of gastric cancer patients and healthy controls ((**a**), Nanosight analyses; (**b**), TEM analyses). **b**, The concentration of exosomes from the serum of gastric cancer patients and healthy controls. **P < 0.01. **c**, The mean diameter of exosomes from the serum of gastric cancer patients and healthy controls. **d**, The size and morphology of exosomes from the culture supernatants of gastric epithelial cells and gastric cancer cells ((**a**), Nanosight analyses; (**b**), TEM analyses). **e**, The concentration of exosomes from the culture supernatants of gastric epithelial cells and gastric cancer cells, **P < 0.01. **f**, The mean diameter of exosomes from the culture supernatants of gastric epithelial cells and gastric cancer cells

The results of transmission electron microscope analysis showed that exosome derived from the serum of gastric cancer patients and healthy controls were dish- or cup-like vesicles with 50–100 nm in diameter and double lipid layer (Fig. 1a). The concentration of exosomes from the serum of gastric cancer patients was higher than that from healthy control (*P* < 0.01) (Fig. 1b). The diameter of exosomes from the serum of gastric cancer patients was similar to that from healthy controls (Fig. 1c). In addition, the concentration of exosome from the culture supernatant of gastric cancer cells MGC-803 and SGC-7901 were also higher than that from gastric epithelial cells GES-1 (*P* <0.01 ) (Fig. 1e). However, the diameter of exosome from the culture supernatant of gastric cancer cells MGC-803 and SGC-7901 has no difference with that from gastric epithelial cells GES-1 (Fig. 1f).

### Proteomic analyses of exosomes from the serum of gastric cancer patients

**Fig. 2:**
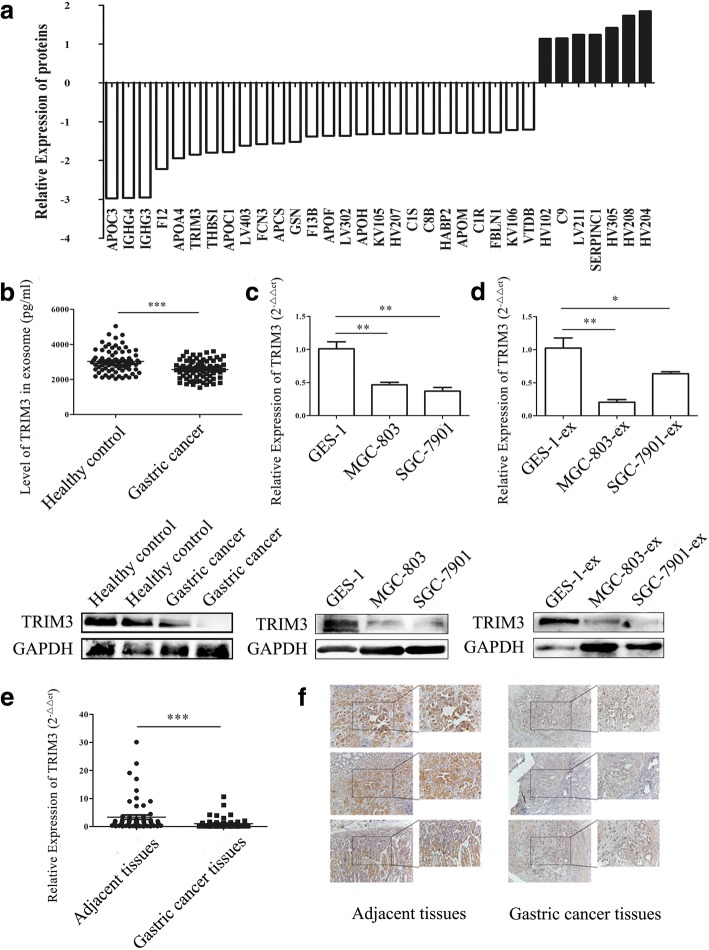
The proteomic analysis of TRIM3 expression in exosomes. **a**, LC-MS/MS analyses of the protein profiles of exosomes from the serum of gastric cancer patients and healthy controls. **b**, ELISA and western blot analyses of the expression of TRIM3 protein in exosomes from the serum of gastric cancer patients and healthy controls, ***P < 0.001. **c**, The expression of TRIM3 gene in gastric epithelial cells and gastric cancer cells was examined by using qRT-PCR and western blot. **P < 0.01. **d**, The expression of TRIM3 gene in exosomes from the culture supernatants of gastric epithelial cells and gastric cancer cells was detected by using qRT-PCR and western blot. *P < 0.05, **P < 0.01. **e**, The expression of TRIM3 gene in gastric cancer tissues and adjacent control tissues was detected by using qRT-PCR. ***P < 0.001. **f**, The expression of TRIM3 protein in gastric cancer tissues and adjacent control tissues was detected by using immunohistochemistry

Three pairs of serum samples were collected from gastric cancer patients and healthy controls and the isolated exosomes from the serum samples were used for LC-MS/MS analyses. A total of 243 differentially expressed proteins were identified in the serum exosomes of gastric cancer patients, of which 33 proteins (7 upregulated and 26 downregulated) showed significant difference between gastric cancer patients and healthy controls (*P* < 0.05) (Fig. 2a) (Additional file 4: Figure S1).

### TRIM3 is downregulated in gastric cancer

We next verified the expression of TRIM3 in gastric cancer tissues and serum exosomes. We performed ELISA to detect TRIM3 expression in exosomes that were extracted from 80 pairs of gastric cancer patients and healthy controls. The results showed that the expression of TRIM3 in exosomes from the serum of gastric cancer patients was much lower than that from the healthy controls, which was further confirmed by western blot assay (Fig. 2b). We next determined the expression of TRIM3 in gastric cancer tissues and adjacent control tissues by using real-time quantitative reverse transcription PCR (qRT-PCR). We found that the expression of TRIM3 was lower in gastric cancer tissues than the paired control tissues in most cases (Fig. 2e). We then conducted immunohistochemistry (IHC) to examine TRIM3 expression in 20 pairs of gastric cancer tissues and adjacent control tissues. We found that TRIM3 was detectable in 15% (3/20) of the gastric cancer tissues and 75% (15/20) of adjacent control tissues (Fig. 2f). The positive rate of TRIM3 was much lower in tumor tissues than adjacent control tissues. We detected the expression of TRIM3 in gastric cancer cells and gastric epithelial cells. TRIM3 protein levels were much lower in MGC-803 and SGC-7901 cells than in GES-1 cells. The decreased expression of TRIM3 was also observed in exosome from the culture supernatant of MGC-803 and SGC-7901 cells compared to that from GES-1 cells (Fig. 2c, d).

### TRIM3 overexpression inhibits the proliferation and migration of gastric cancer cells in vitro

**Fig. 3:**
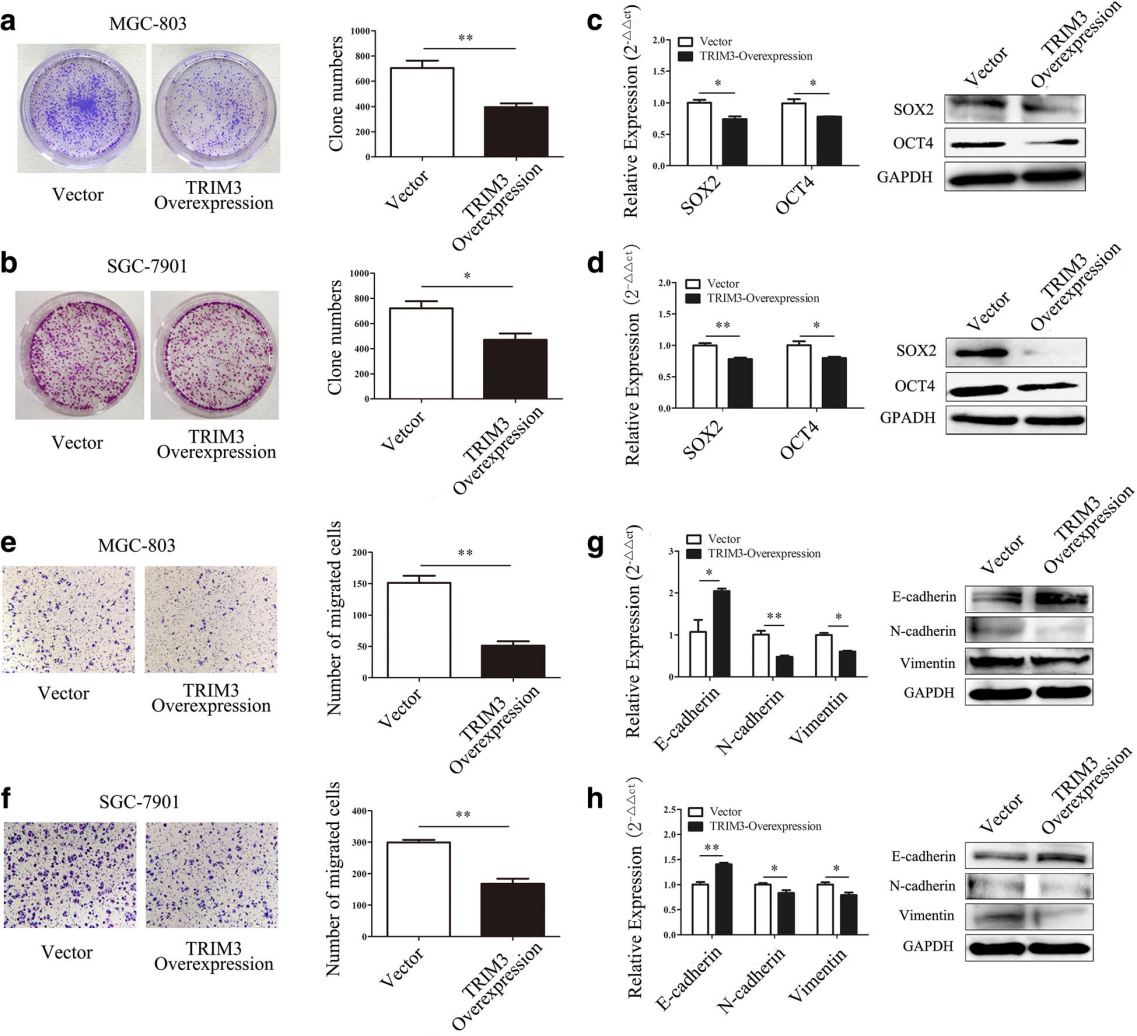
TRIM3 overexpression inhibits gastric cancer cell proliferation and migration in vitro*.* **a** and **b**, TRIM3 overexpression inhibits the colony formation abilities of MGC-803 (**a**) and SGC-7901 (**b**) cells. *P < 0.05, **P < 0.01. **c** and **d**, The expression of SOX2 and OCT4 in gastric cancer cells transfected with TRIM3, **c**, MGC-803; **d**, SGC-7901. *P < 0.05, **P < 0.01. **e** and **f**, TRIM3 overexpression inhibits the migration of MGC-803 (**e**) and SGC-7901 (**f**) cells. Magnification, 100×; **P < 0.01. **g** and **h**, The expression of EMT associated genes in MGC-803 (**g**) and SGC-7901 (**h**) cells transfected with TRIM3. *P < 0.05, **P < 0.01

We next determined the roles of TRIM3 in gastric cancer. TRIM3 was overexpressed in gastric cancer cells by transient transfection. The results of qRT-PCR and western blot confirmed the increase of TRIM3 expression after transfection (Additional file 5: Figure S2a, b). As shown in Fig. 3a and b, TRIM3 overexpression reduced colony formation ability in MGC-803 and SGC-7901cells. TRIM3 overexpression decreased the expression of OCT4 and SOX2, two factors that have been reported to play important roles in cell proliferation, in MGC-803 (*P* < 0.05, *P* < 0.05) and SGC-7901 cells (*P* < 0.01, *P* < 0.05) (Fig. 3c, d). The overexpression of TRIM3 inhibited cell migration in both MGC-803 and SGC-7901 cells (Fig. 3e, f). Overexpression of TRIM3 could markedly upregulate E-cadherin (*P* < 0.05, *P* < 0.01) expression and downregulate N-cadherin (*P* < 0.01, *P* < 0.05) and vimentin (*P* < 0.05, *P* < 0.05) expression (Fig. 3g, h). Taken together, these results suggest that TRIM3 overexpression inhibits the proliferation and migration of MGC-803 and SGC-7901 cells in vitro.

### TRIM3 knockdown promotes the proliferation and migration of gastric cancer cells in vitro

**Fig. 4:**
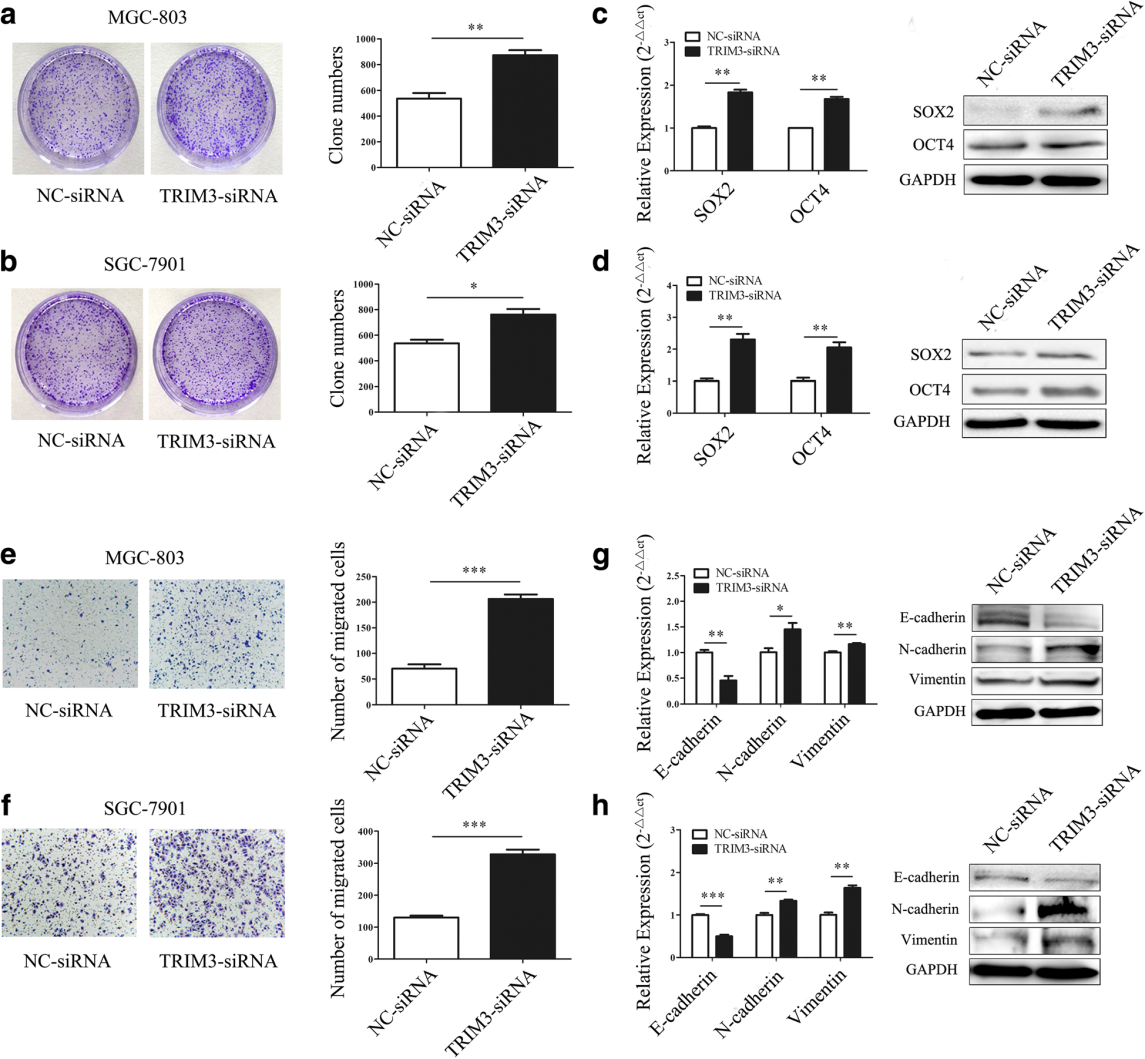
TRIM3 knockdown promotes the proliferation and migration of gastric cancer cells in vitro. **a** and **b**, TRIM3-siRNA promotes the colony formation abilities of MGC-803 (**a**) and SGC-7901 (**b**) cells. *P < 0.05, **P < 0.01. **c** and **d**, The expression of SOX2 and OCT4 in gastric cancer cells transfected with TRIM3-siRNA. (**c**) MGC-803; (**d**) SGC-7901. **P < 0.01. **e** and **f**, TRIM3-siRNA enhances the migration of MGC-803 (**e**) and SGC-7901 (**f**) cells. Magnification 100×; ***P < 0.001. **g** and **h**, The expression of EMT associated genes in MGC-803 (**g**) and SGC-7901 (**h**) cells transfected with TRIM3-siRNA. *P < 0.05, **P < 0.01, ***P < 0.001

We knocked down TRIM3 expression by using siRNA in MGC-803 and SGC-7901 cells (Additional file 5: Figure S2c, d). TRIM3 knockdown cells showed significant increase in colony formation ability compared to scramble control knockdown cells (Fig. 4a, b). The knockdown of TRIM3 markedly upregulated SOX2 (*P* < 0.01, *P* < 0.01) and OCT4 (*P* < 0.01, *P* < 0.01) expression in MGC-803 and SGC-7901 cells (Fig. 4c, d). The results of transwell migration assay showed that TRIM3 knockdown enhanced the migratory abilities of MGC-803 and SGC-7901 cells (Fig. 4e, f). The knockdown of TRIM3 markedly downregulated E-cadherin (*P* < 0.01, *P* < 0.001) expression and upregulated N-cadherin (*P* < 0.05, *P* < 0.01) and vimentin (*P* < 0.01, *P* < 0.01) expression (Fig. 4g, h). Thus, these results suggest that TRIM3 promotes the proliferation and migration of MGC-803 and SGC-7901 in vitro.

### TRIM3 overexpression suppresses gastric cancer growth and metastasis in vivo

**Fig. 5:**
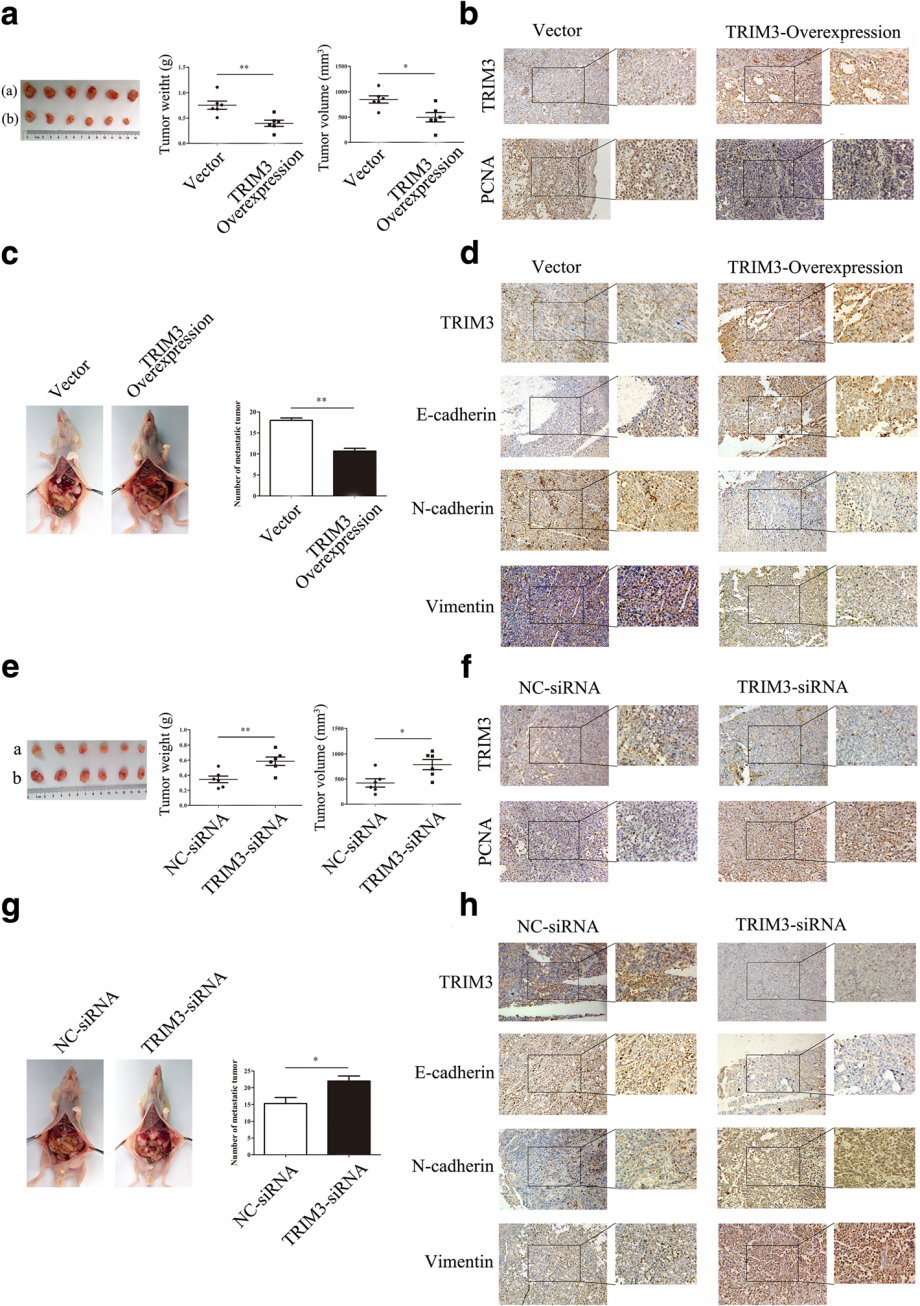
TRIM3 overexpression inhibits gastric cancer growth and metastasis in vivo. **a**, The weight and volumes of tumors in control and TRIM3 overexpression groups. (**(a)**. TRIM3-Vector; (**b**), TRIM3 overexpression). *P < 0.05, ** *P* < 0.01. **b**, The expression of TRIM3 and PCNA in subcutaneous tumor tissues was examined by using immunohistochemistry. Magnification, 200× (left panel); 400× (right panel). **c**, The number of metastatic tumor nodes in control and TRIM3 overexpression groups. **P < 0.01. **d**, The expression of TRIM3 and EMT associated proteins in the metastatic tumor nodes was detected by using immunohistochemistry. Magnification, 200× (left panel); 400× (right panel). **e**, The weight and volume of tumors in control and TRIM3-siRNA groups. *P < 0.01; **P < 0.05. **f**, The expression of TRIM3 and PCNA in subcutaneous tumor tissues was detected by using immunohistochemistry. Magnification, 200× (left panel); 400× (right panel). **g**, The number of metastatic tumor nodes in control and TRIM3-siRNA groups. *P < 0.05. **h**, The expression of TRIM3 and EMT associated proteins in the metastatic tumor nodes was detected by using immunohistochemistry. Magnification, 200× (left panel); 400× (right panel)

To establish the role of TRIM3 as a tumor suppressor in vivo, we used the TRIM3 overexpressing MGC-803 cells to establish xenograft and peritoneal metastasis models in nude mice. In consistent with the in vitro results, the tumor weight and volume were decreased in the TRIM3 transfected MGC-803 group (Fig. 5a) (*P* < 0.01, *P* < 0.05). We found that TRIM3 expression was up-regulated in TRIM3 transfected MGC-803 group by IHC Method (Fig. 5b). Compared with the vector group, the expression of PCNA was decreased in xenograft tumors in the TRIM3 overexpression group (Fig. 5b). We next wanted to know whether TRIM3 overexpression also influenced the metastatic potential of gastric cancer cells in vivo. As shown in Fig. 5c, the number of metastatic tumor nodes in TRIM3 overexpression group was obviously less than that in the vector group (*P* < 0.01). The expression of TRIM3 was up-regulated in TRIM3 transfected MGC-803 group by IHC Method (Fig. 5d). The expression of E-cadherin was increased whereas that of N-cadherin and Vimentin was decreased in the xenograft tumors in the TRIM3 overexpression group (Fig. 5d). Altogether, these results suggest that TRIM3 plays a suppressive role in gastric cancer growth.

### TRIM3 knockdown promotes gastric cancer growth and metastasis in vivo

To investigate whether TRIM3 downregulation could promote gastric cancer growth and metastasis in vivo, we used the MGC-803 cells transfected with TRIM3 siRNA to establish subcutaneous tumor xenograft and peritoneal metastasis models in nude mice. As shown in Fig. 5e, the tumor weight and volume were increased in the TRIM3 siRNA transfected MGC-803 group (*P* < 0.01, *P* < 0.05). We found that TRIM3 expression was down-regulated in TRIM3 knockdown MGC-803 group by IHC Method (Fig. 5f). Compared with the scramble control group, the expression of PCNA was increased in xenograft tumors in the TRIM3 knockdown group (Fig. 5f). We next wanted to know whether TRIM3 downregulation also influenced the metastatic potential of gastric cancer cells in vivo. Representative images of peritoneal metastasis mice were shown in Fig. 5g. The number of metastatic tumor nodes in TRIM3 siRNA group was obviously more than that in the vector group (*P* < 0.05). The expression of TRIM3 was down-regulated in TRIM3 knockdown MGC-803 group by IHC Method (Fig. 5h). The expression of E-cadherin was decreased, whereas that of N-cadherin and Vimentin was increased in the xenograft tumors in the TRIM3 siRNA group compared to the vector group (Fig. 5h). Taken together, these results suggest that TRIM3 downregulation enhances gastric cancer growth and metastasis in vivo.

### Exosomal TRIM3 inhibits the proliferation and migration of gastric cancer cells in vitro

**Fig. 6:**
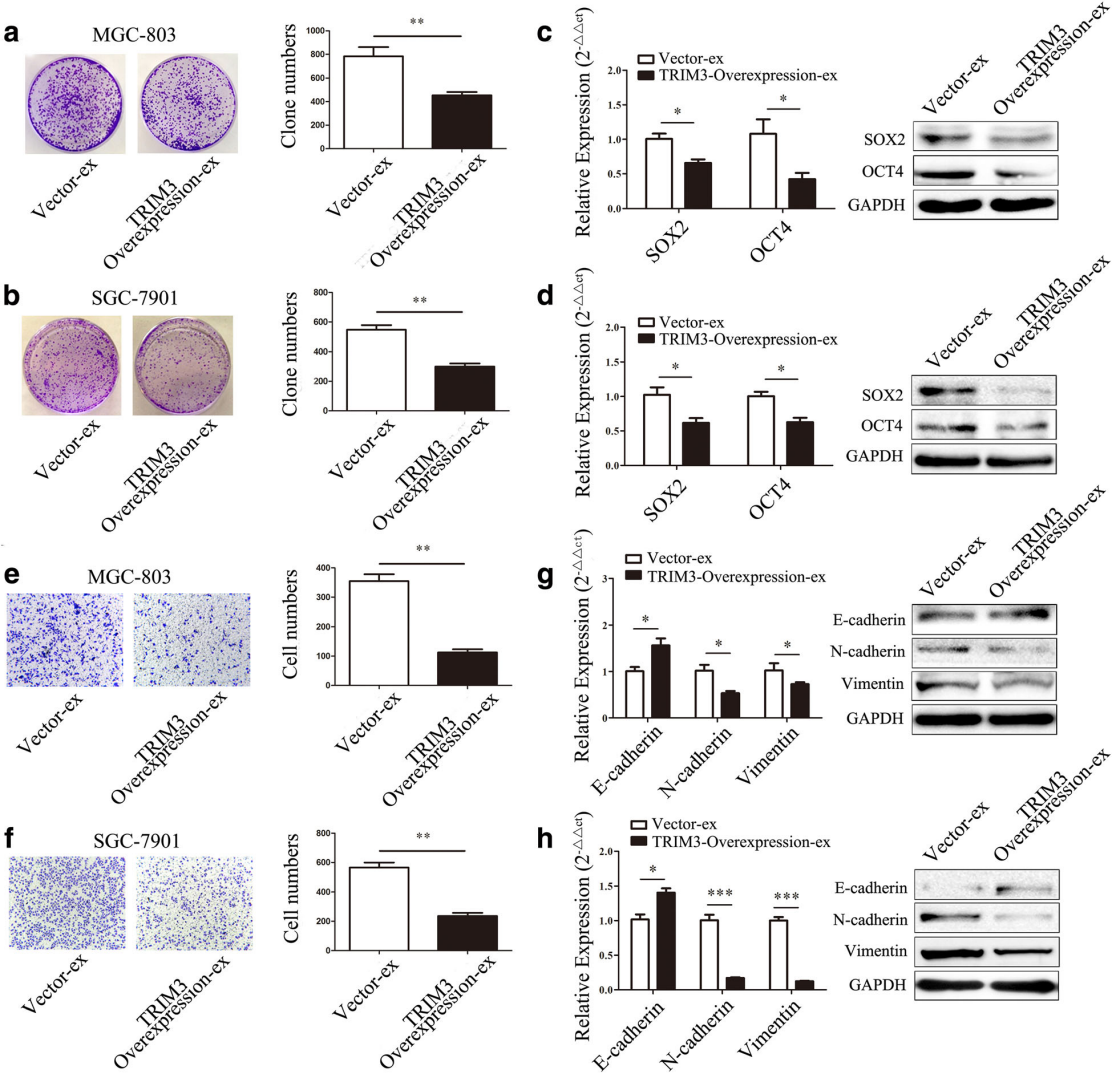
Exosomal TRIM3 inhibits the proliferation and migration of gastric cancer cells in vitro. **a** and **b**, The effects of TRIM3-overexpressing exosomes on the colony formation abilities of MGC-803 and SGC-7901 cells. **P < 0.01. **c** and **d**, The expression of SOX2 and OCT4 genes and proteins in MGC-803 (**c**) and SGC-7901 (**d**) cells treated with 50 μg TRIM3-overexpressing exosomes. *P < 0.05. **e** and **f**, The effects of TRIM3-overexpressing exosomes on the migration of MGC-803 (**e**) and SGC-7901 (**f**) cells. Magnification, 100×; *P < 0.05, **P < 0.01. **g** and **h**, The expression of EMT associated genes and proteins in MGC-803 (**g**) and SGC-7901 (**h**) cells treated with 50 μg TRIM3-overexpressing exosomes. *P < 0.05, ***P < 0.001

To demonstrate that the decreased expression of exosomal TRIM3 is involved in gastric cancer progression, we overexpressed TRIM3 in gastric cancer cells and extracted the TRIM3-overexprressing exosomes from the transfected cells. The expression of TRIM3 was upregulated in exosomes from TRIM3-overexpressing gastric cancer cells (*P* < 0.001) as detected by qRT-PCR and western bolt (Additional file 6: Figure S3). The results of confocal microscope and multispectral imaging flow cytometry showed that the gastric cancer cells efficiently uptook the exosomes that had been labeled with the fluorescent dye DiI (Additional file 7: Figure S4). MGC-803 and SGC-7901 cells were treated with different concentration of TRIM3-overexpressing exosomes (10, 25, 50 μg) for 48 h. The results of western blot assay showed that TRIM3 expression was increased in the gastric cancer cells incubated with the TRIM3-overexpressing exosomes (Additional file 8: Figure S5). We next tested the effects of TRIM3-overexpressing exosomes on gastric cancer cells. The treatment with 50 μg TRIM3-overexpressing exosomes led to a significant decrease in the proliferation and migration of MGC-803 and SGC-7901 cells (Fig. 6a, b and e, f). However, no significant change was observed in gastric cancer cells treated with 10 μg and 25 μg exosomes (Additional file 9: Figure S6). Thus, we chose 50 μg exosome for the following studies. The expression of SOX2 (*P* < 0.05, *P* < 0.05) and OCT4 (*P* < 0.05, *P* < 0.05) in gastric cancer cells treated with 50 μg exosomes were significantly decreased compared to the control group (Fig. 6c, d). We also found that N-cadherin (*P* < 0.05, *P* < 0.001) and vimentin (*P* < 0.05, *P* < 0.001) were downregulated while E-cadherin (*P* < 0.05, *P* < 0.05) was upregulated in 50 μg exosome treated MGC-803 and SGC-7901 cells (Fig. 6g, h). These results suggest that the treatment with 50 μg TRIM3-overexpressing exosomes inhibit the proliferation and migration of gastric cancer cells.

### Exosomal TRIM3 inhibits gastric cancer growth and metastasis in vivo

**Fig. 7:**
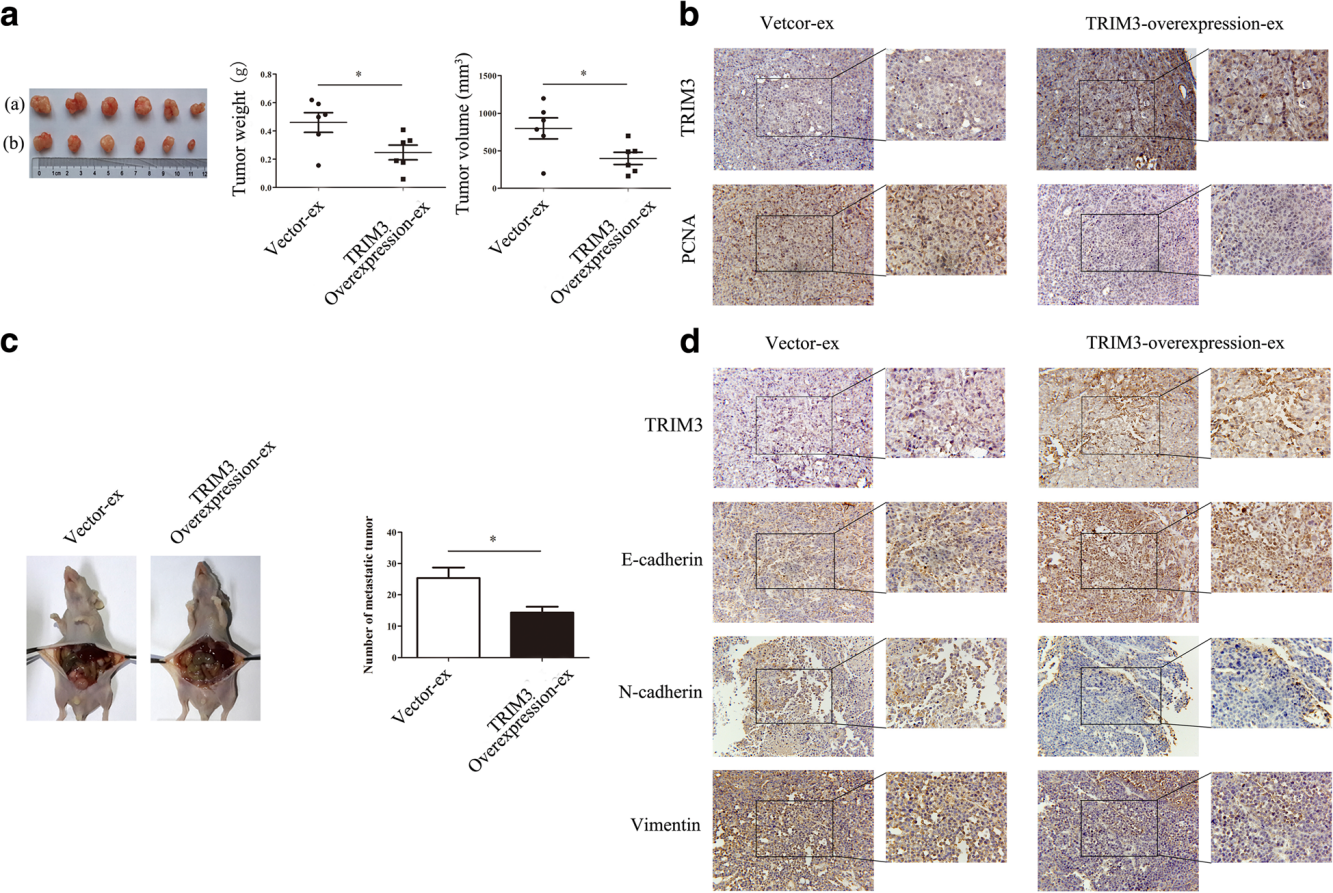
Exosomal TRIM3 inhibits tumor growth and metastasis in vivo. **a**, The effects of TRIM3-overexpressing exosomes on tumor growth in vivo*.* (**(a)**, control exosomes group; (**b**), TRIM3-overexpressing exosome group). *P< 0.05. **b**, The expression of TRIM3 and PCNA in subcutaneous tumor tissues in control exosome and TRIM3-overexpressing exosome groups. Magnification, 200× (left panel); 400× (right panel). **c**, The effects of TRIM3-overexpressing exosomes on tumor metastasis in vivo. The number of metastatic tumor nodes in control exosomes and TRIM3-overexpressing exosome groups were compared. *P < 0.05. **d**, The expression of TRIM3 and EMT associated proteins in the metastatic tumor tissues in control exosome and TRIM3-overexpressing exosome groups. Magnification, 200× (left panel); 400× (right panel)

To determine the effects of TRIM3-overexpressing exosomes on the tumorigenicity and metastasis of gastric cancer, we conducted xenograft tumor and peritoneal metastasis models in nude mice. MGC-803 cells pre-treated with 50 μg TRIM3-overexpression exosomes for 48 h were subcutaneously implanted or intraperitoneal implanted into nude mice. The tumor weight (*P* < 0.05) and volume (*P* < 0.05) in mice injected with MGC-803 cells pre-treated with 50 μg TRIM3-overexpressing exosomes were significantly lower than that in controls (Fig. 7a). We found that TRIM3 expression was up-regulated in xenograft tumors in the TRIM3-overexpressing exosomes treated group by IHC Method (Fig. 7b). Compared with the vector group, the expression of PCNA was decreased in xenograft tumors in the TRIM3-overexpressing exosomes treated group (Fig. 7b). As shown in Fig. 7c, the number of metastatic tumor nodes in 50 μg TRIM3-overexpressing exosomes treated group was obviously less than in the vector group (*P* < 0.05). The expression of TRIM3 was up-regulated in xenograft tumors in the TRIM3-overexpressing exosomes treated group (Fig. 7d). We then find, the expression of E-cadherin was increased whereas that of N-cadherin and Vimentin was decreased in xenograft tumors in the TRIM3-overexpressing exosomes treated group (Fig. 7d). Altogether, these results suggest that TRIM3-overexpressing exosomes could transfer TRIM3 to gastric cancer cells, which inhibit gastric cancer growth and metastasis in vivo.

### TRIM3 downregulation is associated with miR-20a in gastric cancer

**Fig. 8:**
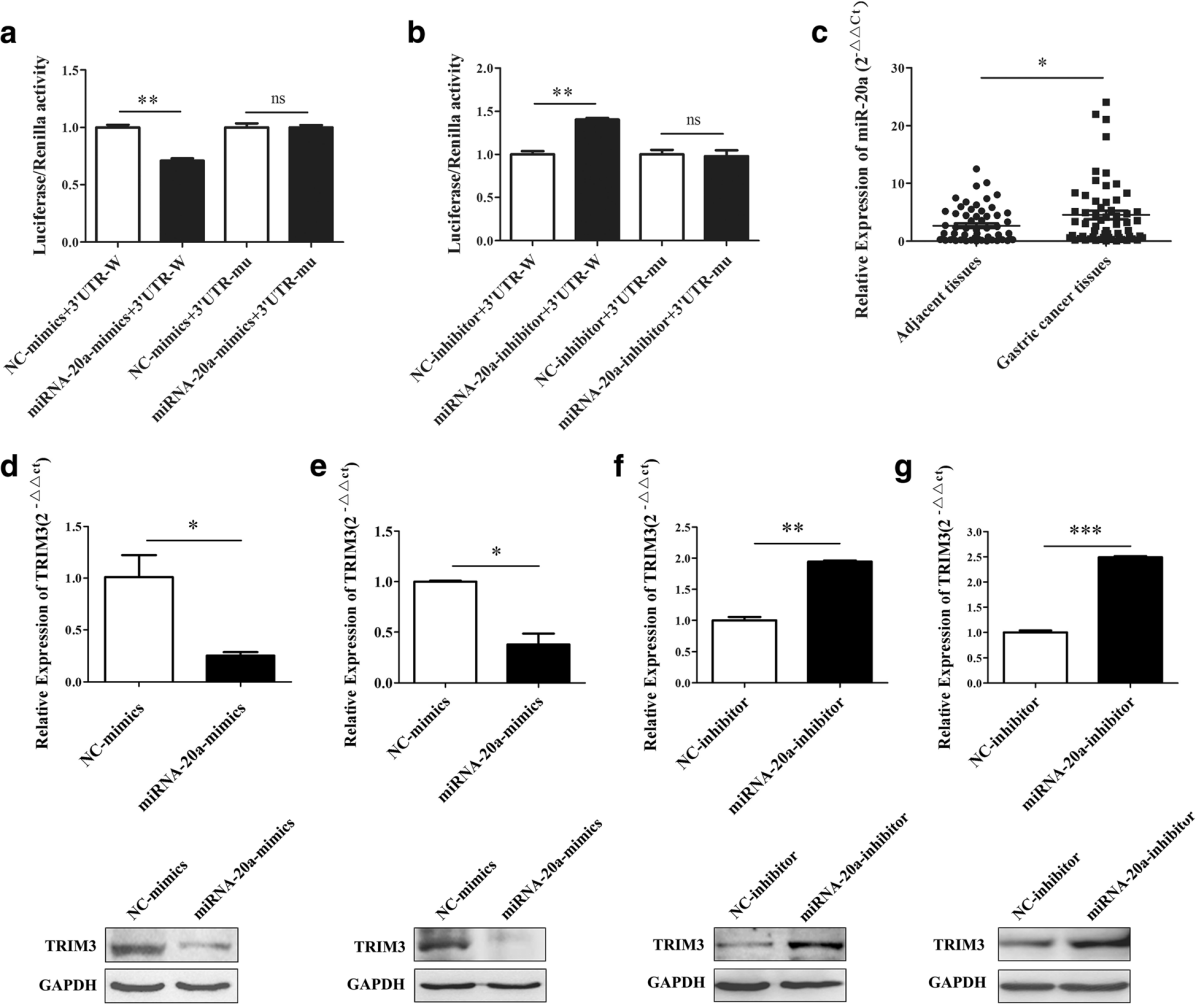
TRIM3 downregulation is associated with miR-20a in gastric cancer. **a**, miR-20a mimics suppressed the luciferase activity of the wild type TRIM3 3’UTR (WT) (*P < 0.01) in HEK-293 cells. **b**, miR-20a-inhibitor enhanced the luciferase activity of the wild type TRIM3 3’UTR (WT) (*P < 0.01) in HEK-293 cells. **c**, miR-20a expression in gastric cancer tissues and adjacent control tissues, *P < 0.05. **d** and e, The expression of TRIM3 gene in miR-20a-mimics transfected MGC-803(d) and SGC-7901(**e**) examined by qRT-PCR and western blot. *P < 0.05. f and g, The expression of TRIM3 gene in miR-20a-inhibitor transfected MGC-803(f) and SGC-7901(**g**) examined by qRT-PCR and western blot. **P < 0.01, ***P < 0.001

To investigate the possible upstream regulator of TRIM3, we used Targetscan to predict TRIM3-targeting miRNAs and found that miR-20a potentially bound to TRIM3 mRNA. To confirm this, we constructed the TRIM3 3’UTR containing the target sequences, or the mutants, into a dual-luciferase reporter vector (Fig. 8a, b). The transfection of miR-20a suppressed the luciferase activity of the wild type TRIM3 3’UTR (WT) (*P* < 0.01), while mutation of the miR-20a binding sites (Mut) reversed this suppression. On the contrary, miR-20a-inhibitor enhanced the luciferase activity of the wild type TRIM3 3’UTR (*P* < 0.01), while mutation of the miR-20a binding sites blocked this enhancement. In addition, the expression of miR-20a was significantly upregulated in gastric cancer tissues compared to the matched control tissues (*P* < 0.01) (Fig. 8c). The level of TRIM3 was negatively correlated with that of miR-20a in gastric cancer tissues. To further confirm the direct effect of miR-20a on TRIM3, we transfect miR-20a-mimics and miR-20a-inhibitor into gastric cancer cell lines. The results of qRT-PCR confirmed the decrease of TRIM3 expression after miR-20a-mimics transfection in MGC-803 (*P* < 0.05) and SGC-7901 (*P* < 0.05), and the protein expression level of TRIM3 were significantly lower than that of control group by western blot (Fig. 8d and e). We also found that TRIM3 were upregulated after miR-20a-inhibitor transfection in MGC-803 (*P* < 0.01) and SGC-7901 (*P* < 0.001), the results of western blot showed that miR-20a-inhibitor transfection enhanced the expression of TRIM3 in MGC-803 and SGC-7901 cells (Fig. 8f and g). Thus, these results suggest that TRIM3 is the direct target of miR-20a, TRIM3 was negatively associated with miRNA-20a, and directly regulated by miR-20a.

## Discussion

Cancer cells release excessive quantities of exosomes. The plasma of patients with acute myeloid leukemia (AML) were found to contain a 60-fold greater quantity of exosomes than that of normal donors [19]. Melanoma cells also release large quantities of exosomes, in contrast to normal melanocytes [20, 21]. Patients with ovarian cancer exhibit significantly increased levels of serum exosomes compared to benign disease or healthy controls [22]. Silva et al. have reported that high levels of exosomes in the plasma of colorectal cancer patients were significantly associated with poorly differentiated tumors and with decreased overall survival [23]. These reports indicate that the level of exosomes is elevated in the circulation of cancer patients and is positively associated with disease progression. In this study, we found that the concentration of exosomes from the serum of gastric cancer patients was higher than that from the healthy controls, which is consistent with that reported in the other cancers, suggesting that the increased level of exosomes is an important indicator of cancer progression.

Exosomes perform a variety of extracellular functions in the processes of cancer development and progression [24, 25]. Exosomes contain a wide range of functional proteins and nucleic acids that can be transferred to local and distant recipient cells [26–29]. Tumor cells secrete large amounts of exosomes that promote tumor progression through communication between the tumor and surrounding stromal tissue, activation of proliferative and angiogenic pathways, and initiation of premetastatic sites [30–34]. Therefore, exosomes are an important component of the tumor microenvironment and are currently considered to be one of the main contributors to tumor progression and metastasis [24]. In this study, we initially purified exosomes from the serum of gastric cancer patients and explored the protein profiles of exosomes by using LC-MS/MS. We identified a total of 243 differentially expressed proteins between the exosomes from the serum of gastric cancer patients and that from the serum of healthy controls. The altered expression of proteins in exosomes may act as messengers that transport signals between cells to promote cancer progression.

At present, there are few studies about the role of TRIM3 in cancer. It has been reported that the mRNA and protein levels of TRIM3 are downregulated in hepatocellular carcinoma (HCC) and are correlated with an unfavorable prognosis in patients with HCC, suggesting that TRIM3 could be a prognostic marker and novel therapeutic target for HCC [35]. Liu et al. have reported that TRIM3 is a tumor suppressor mapping to chromosome 11p15.5 and that it might block tumor growth by sequestering p21 and preventing it from facilitating the accumulation of cyclin D1-cdk4 [17]. Chen et al. have shown that TRIM3 acts as a tumor suppressor in GBM by restoring asymmetric cell division [16]. Boulay et al. have reported the homozygous deletions of TRIM3 in brain tumor [36]. Piao et al. suggest that TRIM3 functions as a tumor suppressor in colorectal cancer (CRC), which is exerted partially through the regulation of p53 [37]. We found that TRIM3 was significantly downregulated in gastric cancer tissues. We investigated the functional roles of TRIM3 in gastric cancer by using gain- and loss-of-function studies. We found that TRIM3 inhibited gastric cancer growth and metastasis through the regulation of stem cell factors and EMT regulators, indicating that TRIM3 also plays tumor suppressive roles in gastric cancer. However, more studies are warranted to clarify the molecular mechanism responsible for the roles of TRIM3 in gastric cancer. We further identified TRIM3 as a direct target of miR-20a, and negatively regulated by miR-20a. MiR-20a act as an oncogenic miRNA that has been previously reported to be overexpressed in most cancers, suggesting that the upregulated expression of miRNAs in cancer may be associated with the decreased expression of TRIM in gastric cancer.

Exosomes contain bioactive molecules that reflect the pathological state of the originated cells, thus providing an enriched source of biomarkers [38–40]. Melo et al. have reported that the cell surface proteoglycan glypican-1 can be detected on exosomes harvested from the serum of patients with pancreatic cancer and breast cancer [41]. EBV-positive nasopharyngeal carcinoma (NPC) cell-derived exosomes contain HIF-1α, which increases the migration and invasiveness of EBV-negative NPC cells [42]. In this study, we demonstrated that the expression of TRIM3 was lowly expressed in exosomes from the serum of gastric cancer patients and from the culture supernatants of gastric cancer cells, suggesting that the decreased expression of TRIM3 in serum exosomes could be a diagnostic biomarker for gastric cancer.

The tumor suppressive role of TRIM3 in gastric cancer and the presence of TRIM3 in exosomes led us to explore the possibility of delivering TRIM3 by exosomes to treat gastric cancer. We overexpressed TRIM3 in gastric cancer cells and collected the TRIM3-overexpressing exosomes and incubated gastric cancer cells with the TRIM3-overexpressing exosomes. We found that the TRIM3-overexpressing exosomes could be internalized into the gastric cancer cells. Exosomal TRIM3 could inhibit gastric cancer growth and metastasis in vitro and in vivo, suggesting that exosomes-mediated transfer of TRIM3 might serve as a new strategy for gastric cancer therapy.

## Conclusions

Our findings suggest that TRIM3 expression is downregulated in gastric cancer. TRIM3 functions as a tumor suppressor by inhibiting gastric cancer growth and metastasis. TRIM3 could be a utilized as a diagnostic biomarker and therapeutic target for gastric cancer.

## Additional files

Additional file 1: **Table S1.** Sequences of TRIM3-siRNA. (DOCX 18 kb)

Additional file 2: **Table S2.** Primer sequences and amplified fragment products. (DOCX 19 kb)

Additional file 3: **Table S3.** Sequences of miR-20a mimics and inhibitor. (DOCX 18 kb)

Additional file 4: **Figure S1.** Analysis of the proteins in exosome derived from serum of gastric cancer patients and healthy control. (JPG 454 kb)

Additional file 5: **Figure S2.** Validation of TRIM3 expression levels in TRIM3 overexpression and knockdown cells. a and b, The expression of TRIM3 in MGC-803 and SGC-7901 cells transfected with TRIM3 was detected by using qRT-PCR and western blot. ***P < 0.001. c and d, The expression of TRIM3 in MGC-803 and SGC-7901 transfected with TRIM3-siRNA was determined by using qRT-PCR and western blot. ***P < 0.001. (JPG 607 kb)

Additional file 6: **Figure S3.** The expression of TRIM3 in exosomes from gastric cancer cells transfected with TRIM3. a, MGC-803; b, SGC-7901; ***P < 0.001. (JPG 259 kb)

Additional file 7: **Figure S4.** Gastric cancer cells internalize exosomes. a, Exosome internalization into gastric cancer cells was determined by using confocal microscopy. Magnification, 200× ((a) and (b)); 400× ((c) and (d)). b, Exosome internalization into gastric cancer cells was determined by using imaging flow cytometry. (JPG 3089 kb)

Additional file 8: **Figure S5.** The expression of TRIM3 in gastric cancer cells treated with control exosomes and TRIM3-overexpressing exosomes. (JPG 130 kb)

Additional file 9: **Figure S6.** The effects of TRIM3-overexpressing exosomes on the proliferation and migration of gastric cancer cells in vitro. a and b, The effects of TRIM3-overexpressing exosomes on the colony formation abilities of MGC-803 (a) and SGC-7901 (b) cells. **P < 0.01. c and d, The effects of TRIM3-overexpressing exosomes on the migration of MGC-803 (c) and SGC-7901 (d) cells. *P < 0.05, **P < 0.01. (JPG 5700 kb)

## Abbreviations

ELISA: Enzyme-linked immunosorbent assay
EMT: Epithelial-mesenchymal transition
ex: Exosome
GC: Gastric cancer
IHC: Immunohistochemistry
miR-20a: miRNA-20a.
MVBs: Multivesicular bodies
TRIM: Tripartite motif
TRIM3: Tripartite motif-containing 3

## References

[1] Jemal A, Siegel R, Ward E, Murray T, Xu J, Smigal C, Thun MJ (2006). Cancer statistics, 2006. CA Cancer J Clin.

[2] Saka M, Morita S, Fukagawa T, Katai H (2011). Present and future status of gastric cancer surgery. Jpn J Clin Oncol.

[3] Boukouris S, Mathivanan S (2015). Exosomes in bodily fluids are a highly stable resource of disease biomarkers. Proteomics Clin Appl.

[4] Thery C, Ostrowski M, Segura E (2009). Membrane vesicles as conveyors of immune responses. Nat Rev Immunol.

[5] Kowal J, Tkach M, Thery C (2014). Biogenesis and secretion of exosomes. Curr Opin Cell Biol.

[6] Vlassov AV, Magdaleno S, Setterquist R, Conrad R (2012). Exosomes: current knowledge of their composition, biological functions, and diagnostic and therapeutic potentials. Biochim Biophys Acta.

[7] Duijvesz D, Luider T, Bangma CH, Jenster G (2011). Exosomes as biomarker treasure chests for prostate cancer. Eur Urol.

[8] Hessvik NP, Sandvig K, Llorente A (2013). Exosomal miRNAs as biomarkers for prostate cancer. Front Genet.

[9] Rabinowits G, Gercel-Taylor C, Day JM, Taylor DD, Kloecker GH (2009). Exosomal microRNA: a diagnostic marker for lung cancer. Clin Lung Cancer.

[10] Bard MP, Hegmans JP, Hemmes A, Luider TM, Willemsen R, Severijnen LA, van Meerbeeck JP, Burgers SA, Hoogsteden HC, Lambrecht BN (2004). Proteomic analysis of exosomes isolated from human malignant pleural effusions. Am J Respir Cell Mol Biol.

[11] Street JM, Barran PE, Mackay CL, Weidt S, Balmforth C, Walsh TS, Chalmers RT, Webb DJ, Dear JW (2012). Identification and proteomic profiling of exosomes in human cerebrospinal fluid. J Transl Med.

[12] Hatakeyama S (2011). TRIM proteins and cancer. Nat Rev Cancer.

[13] Cambiaghi V, Giuliani V, Lombardi S, Marinelli C, Toffalorio F, Pelicci PG (2012). TRIM proteins in cancer. Adv Exp Med Biol.

[14] El-Husseini AE, Vincent SR (1999). Cloning and characterization of a novel RING finger protein that interacts with class V myosins. J Biol Chem.

[15] El-Husseini AE, Kwasnicka D, Yamada T, Hirohashi S, Vincent SR (2000). BERP, a novel ring finger protein, binds to alpha-actinin-4. Biochem Biophys Res Commun.

[16] Chen G, Kong J, Tucker-Burden C, Anand M, Rong Y, Rahman F, Moreno CS, Van Meir EG, Hadjipanayis CG, Brat DJ (2014). Human brat ortholog TRIM3 is a tumor suppressor that regulates asymmetric cell division in glioblastoma. Cancer Res.

[17] Liu Y, Raheja R, Yeh N, Ciznadija D, Pedraza AM, Ozawa T, Hukkelhoven E, Erdjument-Bromage H, Tempst P, Gauthier NP (2014). TRIM3, a tumor suppressor linked to regulation of p21(Waf1/Cip1). Oncogene.

[18] Raheja R, Liu Y, Hukkelhoven E, Yeh N, Koff A (2014). The ability of TRIM3 to induce growth arrest depends on RING-dependent E3 ligase activity. Biochem J.

[19] Szczepanski MJ, Szajnik M, Welsh A, Whiteside TL, Boyiadzis M (2011). Blast-derived microvesicles in sera from patients with acute myeloid leukemia suppress natural killer cell function via membrane-associated transforming growth factor-beta1. Haematologica.

[20] Felicetti F, Parolini I, Bottero L, Fecchi K, Errico MC, Raggi C, Biffoni M, Spadaro F, Lisanti MP, Sargiacomo M, Care A (2009). Caveolin-1 tumor-promoting role in human melanoma. Int J Cancer.

[21] Xiao D, Ohlendorf J, Chen Y, Taylor DD, Rai SN, Waigel S, Zacharias W, Hao H, McMasters KM (2012). Identifying mRNA, microRNA and protein profiles of melanoma exosomes. PLoS One.

[22] Taylor DD, Gercel-Taylor C (2008). MicroRNA signatures of tumor-derived exosomes as diagnostic biomarkers of ovarian cancer. Gynecol Oncol.

[23] Silva J, Garcia V, Rodriguez M, Compte M, Cisneros E, Veguillas P, Garcia JM, Dominguez G, Campos-Martin Y, Cuevas J (2012). Analysis of exosome release and its prognostic value in human colorectal cancer. Genes Chromosomes Cancer.

[24] Peinado H, Lavotshkin S, Lyden D (2011). The secreted factors responsible for pre-metastatic niche formation: old sayings and new thoughts. Semin Cancer Biol.

[25] O'Loughlin AJ, Woffindale CA, Wood MJ (2012). Exosomes and the emerging field of exosome-based gene therapy. Curr Gene Ther.

[26] Mathivanan S, Ji H, Simpson RJ (2010). Exosomes: extracellular organelles important in intercellular communication. J Proteome.

[27] Valadi H, Ekstrom K, Bossios A, Sjostrand M, Lee JJ, Lotvall JO (2007). Exosome-mediated transfer of mRNAs and microRNAs is a novel mechanism of genetic exchange between cells. Nat Cell Biol.

[28] Skog J, Wurdinger T, van Rijn S, Meijer DH, Gainche L, Sena-Esteves M, Curry WJ, Carter BS, Krichevsky AM, Breakefield XO (2008). Glioblastoma microvesicles transport RNA and proteins that promote tumour growth and provide diagnostic biomarkers. Nat Cell Biol.

[29] Martins VR, Dias MS, Hainaut P (2013). Tumor-cell-derived microvesicles as carriers of molecular information in cancer. Curr Opin Oncol.

[30] King HW, Michael MZ, Gleadle JM (2012). Hypoxic enhancement of exosome release by breast cancer cells. BMC Cancer.

[31] Webber J, Steadman R, Mason MD, Tabi Z, Clayton A (2010). Cancer exosomes trigger fibroblast to myofibroblast differentiation. Cancer Res.

[32] Liu C, Yu S, Zinn K, Wang J, Zhang L, Jia Y, Kappes JC, Barnes S, Kimberly RP, Grizzle WE, Zhang HG (2006). Murine mammary carcinoma exosomes promote tumor growth by suppression of NK cell function. J Immunol.

[33] Hood JL, San RS, Wickline SA (2011). Exosomes released by melanoma cells prepare sentinel lymph nodes for tumor metastasis. Cancer Res.

[34] Peinado H, Aleckovic M, Lavotshkin S, Matei I, Costa-Silva B, Moreno-Bueno G, Hergueta-Redondo M, Williams C, Garcia-Santos G, Ghajar C (2012). Melanoma exosomes educate bone marrow progenitor cells toward a pro-metastatic phenotype through MET. Nat Med.

[35] Chao J, Zhang XF, Pan QZ, Zhao JJ, Jiang SS, Wang Y, Zhang JH, Xia JC (2014). Decreased expression of TRIM3 is associated with poor prognosis in patients with primary hepatocellular carcinoma. Med Oncol.

[36] Boulay JL, Stiefel U, Taylor E, Dolder B, Merlo A, Hirth F (2009). Loss of heterozygosity of TRIM3 in malignant gliomas. BMC Cancer.

[37] Piao MY, Cao HL, He NN, Xu MQ, Dong WX, Wang WQ, Wang BM, Zhou B (2016). Potential role of TRIM3 as a novel tumour suppressor in colorectal cancer (CRC) development. Scand J Gastroenterol.

[38] Al-Nedawi K, Meehan B, Micallef J, Lhotak V, May L, Guha A, Rak J (2008). Intercellular transfer of the oncogenic receptor EGFRvIII by microvesicles derived from tumour cells. Nat Cell Biol.

[39] Demory BM, Higginbotham JN, Franklin JL, Ham AJ, Halvey PJ, Imasuen IE, Whitwell C, Li M, Liebler DC, Coffey RJ (2013). Proteomic analysis of exosomes from mutant KRAS colon cancer cells identifies intercellular transfer of mutant KRAS. Mol Cell Proteomics.

[40] Costa-Silva B, Aiello NM, Ocean AJ, Singh S, Zhang H, Thakur BK, Becker A, Hoshino A, Mark MT, Molina H (2015). Pancreatic cancer exosomes initiate pre-metastatic niche formation in the liver. Nat Cell Biol.

[41] Melo SA, Luecke LB, Kahlert C, Fernandez AF, Gammon ST, Kaye J, LeBleu VS, Mittendorf EA, Weitz J, Rahbari N (2015). Glypican-1 identifies cancer exosomes and detects early pancreatic cancer. Nature.

[42] Aga M, Bentz GL, Raffa S, Torrisi MR, Kondo S, Wakisaka N, Yoshizaki T, Pagano JS, Shackelford J (2014). Exosomal HIF1alpha supports invasive potential of nasopharyngeal carcinoma-associated LMP1-positive exosomes. Oncogene.

