# Supplementary material for: Exosomal TRIM3 is a novel marker and therapy target for gastric cancer
Source: J Exp Clin Cancer Res. 2018 Jul 21;37:162. doi: 10.1186/s13046-018-0825-0 (PMC6054744; doi:10.1186/s13046-018-0825-0)
Supplement: Supplementary file 1 — Table S1. Sequences of TRIM3-siRNA. (DOCX 18 kb) [file 13046_2018_825_MOESM1_ESM.docx]

**Additional file Table S1. Sequences of TRIM3-siRNA**

| Gene |  | Sequence（5'- 3'） |
| --- | --- | --- |
| TRIM3-siRNA | Sense | CCACAAGAAUGGCACAUAUTT |
|  | Antisense | AUAUGUGCCAUUCUUGUGGTT |
| NC-siRNA | Sense | UUCUCCGAACGUGUCACGUTT |
|  | Antisense | ACGUGACACGUUCGGAGAATT |
