# Supplementary material for: Exosomal TRIM3 is a novel marker and therapy target for gastric cancer
Source: J Exp Clin Cancer Res. 2018 Jul 21;37:162. doi: 10.1186/s13046-018-0825-0 (PMC6054744; doi:10.1186/s13046-018-0825-0)
Supplement: Supplementary file 2 — Table S2. Primer sequences and amplified fragment products. (DOCX 19 kb) [file 13046_2018_825_MOESM2_ESM.docx]

**Additional file Table S2. Primer sequences and amplified fragment products**

| Gene | Sequence（5'- 3'） | Annealing Temperature | Length |
| --- | --- | --- | --- |
| TRIM3 | F：GCCAATGGACAAGCAGTTC | 60^o^C | 150bp |
|  | R：CGGCATACTGGACAGGATA |  |  |
| SOX2 | F：TTGAGGCTCTGCAGCTTAG | 60^o^C | 285bp |
|  | R：GCCGGTTACAGAACCACAC |  |  |
| OCT4 | F：ACACCAATCCCATCCACACT | 60^o^C | 224bp |
|  | R：GCAAACTTCCTGCAAAGCTC |  |  |
| E-cadherin | F：CGCATTGCCACATACACTCT | 60^o^C | 252bp |
|  | R：TTGGCTGAGGATGGTGTAAG |  |  |
| N-cadherin | F：AGTCAACTGCAACCGTGTCT | 60^o^C | 337bp |
|  | R：AGCGTTCCTGTTCCACTCAT |  |  |
| Vimentin | F：GAGCTGCAGGAGCTGAATG | 60^o^C | 344bp |
|  | R：AGGTCAAGACGTGCCAGAG |  |  |
| β-actin | F：CACGAAACTACCTTCAACTCC | 60^o^C | 265bp |
|  | R：CATACTCCTGCTTGCTGATC |  |  |
