# Supplementary material for: Exosomal TRIM3 is a novel marker and therapy target for gastric cancer
Source: J Exp Clin Cancer Res. 2018 Jul 21;37:162. doi: 10.1186/s13046-018-0825-0 (PMC6054744; doi:10.1186/s13046-018-0825-0)
Supplement: Supplementary file 3 — Table S3. Sequences of miR-20a mimics and inhibitor. (DOCX 18 kb) [file 13046_2018_825_MOESM3_ESM.docx]

**Additional file Table S3. Sequences of miR-20a mimics and inhibitor**

| Gene | Sequence（5'- 3'） |
| --- | --- |
| miR-20a-mimics | UAAAGUGCUUAUAGUGCAGGUAG |
|  | ACCUGCACUAUAAGCACUUUAUU |
| NC-mimics | UUCUCCGAACGUGUCACGUTT |
|  | ACGUGACACGUUCGGAGAATT |
| miR-20a-inhibitor | CUACCUGCACUAUAAGCACUUUA |
| NC-inhibitor | CAGUACUUUUGUGUAGUACAA |
