# Supplementary figures and images for: Exosomal TRIM3 is a novel marker and therapy target for gastric cancer
Source: J Exp Clin Cancer Res. 2018 Jul 21;37:162. doi: 10.1186/s13046-018-0825-0 (PMC6054744; doi:10.1186/s13046-018-0825-0)

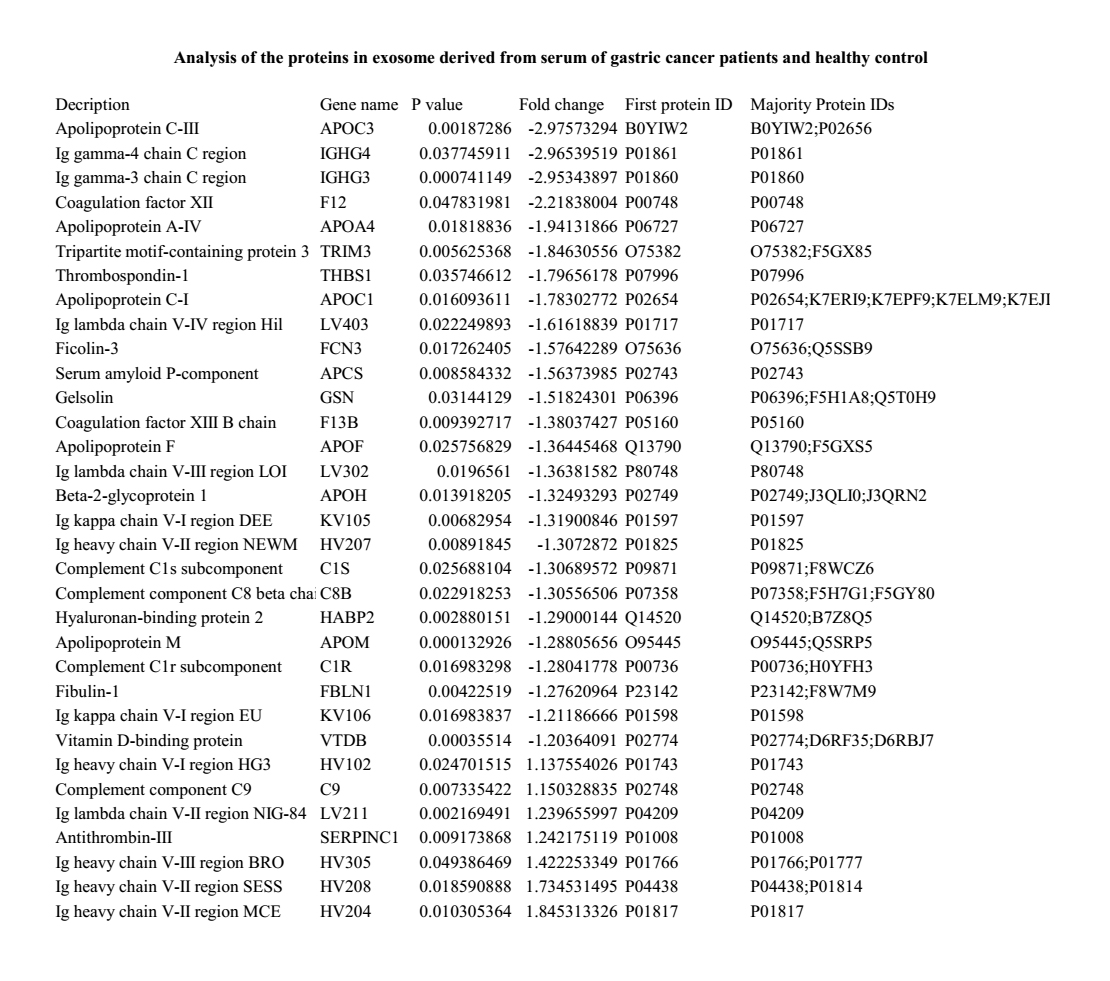

Supplement: Supplementary file 4 — Figure S1. Analysis of the proteins in exosome derived from serum of gastric cancer patients and healthy control. (JPG 454 kb) [file 13046_2018_825_MOESM4_ESM.jpg]

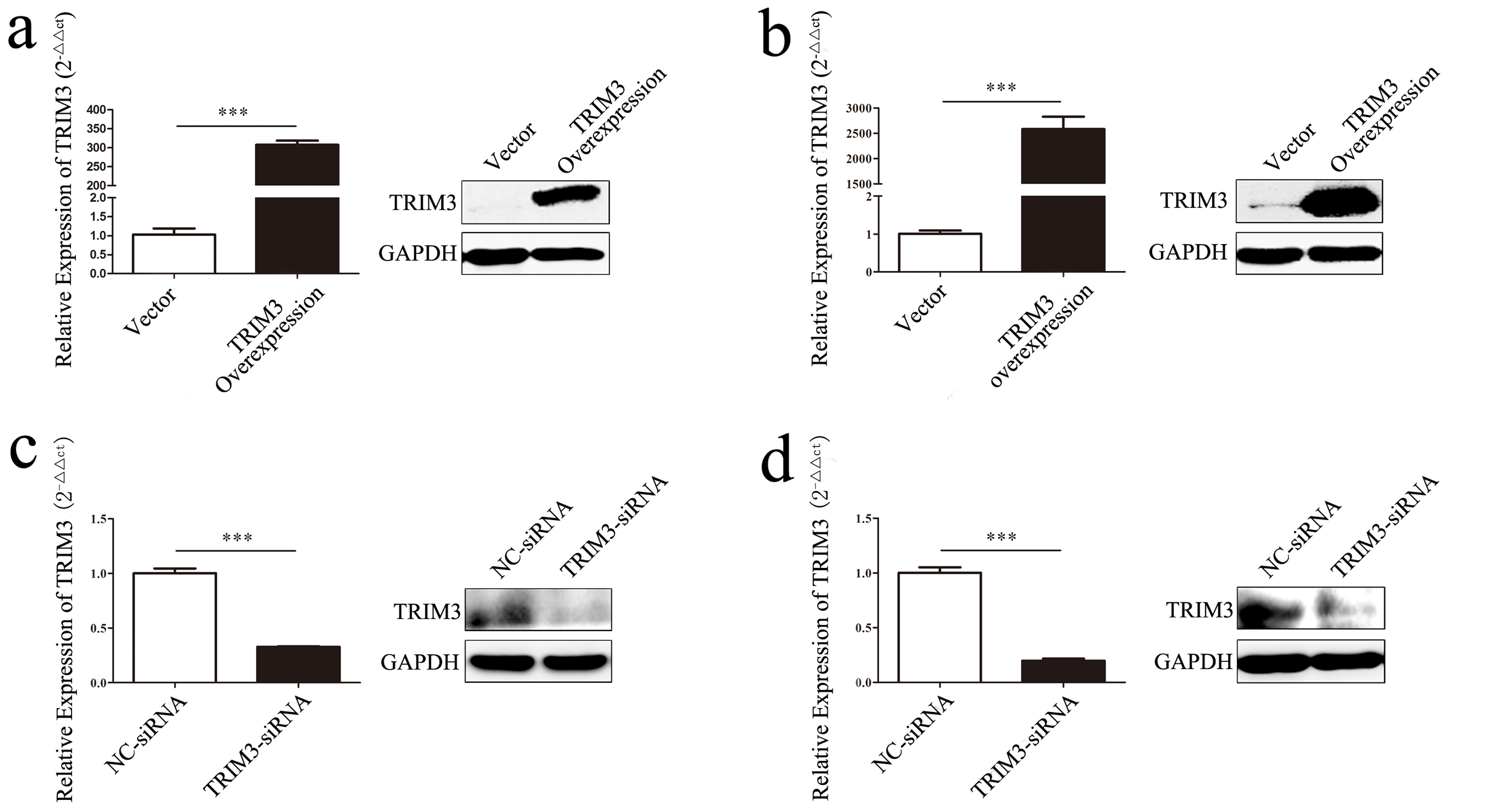

Supplement: Supplementary file 5 — Figure S2. Validation of TRIM3 expression levels in TRIM3 overexpression and knockdown cells. a and b, The expression of TRIM3 in MGC-803 and SGC-7901 cells transfected with TRIM3 was detected by using qRT-PCR and western blot. ***P < 0.001. c and d, The expression of TRIM3 in MGC-803 and SGC-7901 transfected with TRIM3-siRNA was determined by using qRT-PCR and western blot. ***P < 0.001. (JPG 607 kb) [file 13046_2018_825_MOESM5_ESM.jpg]

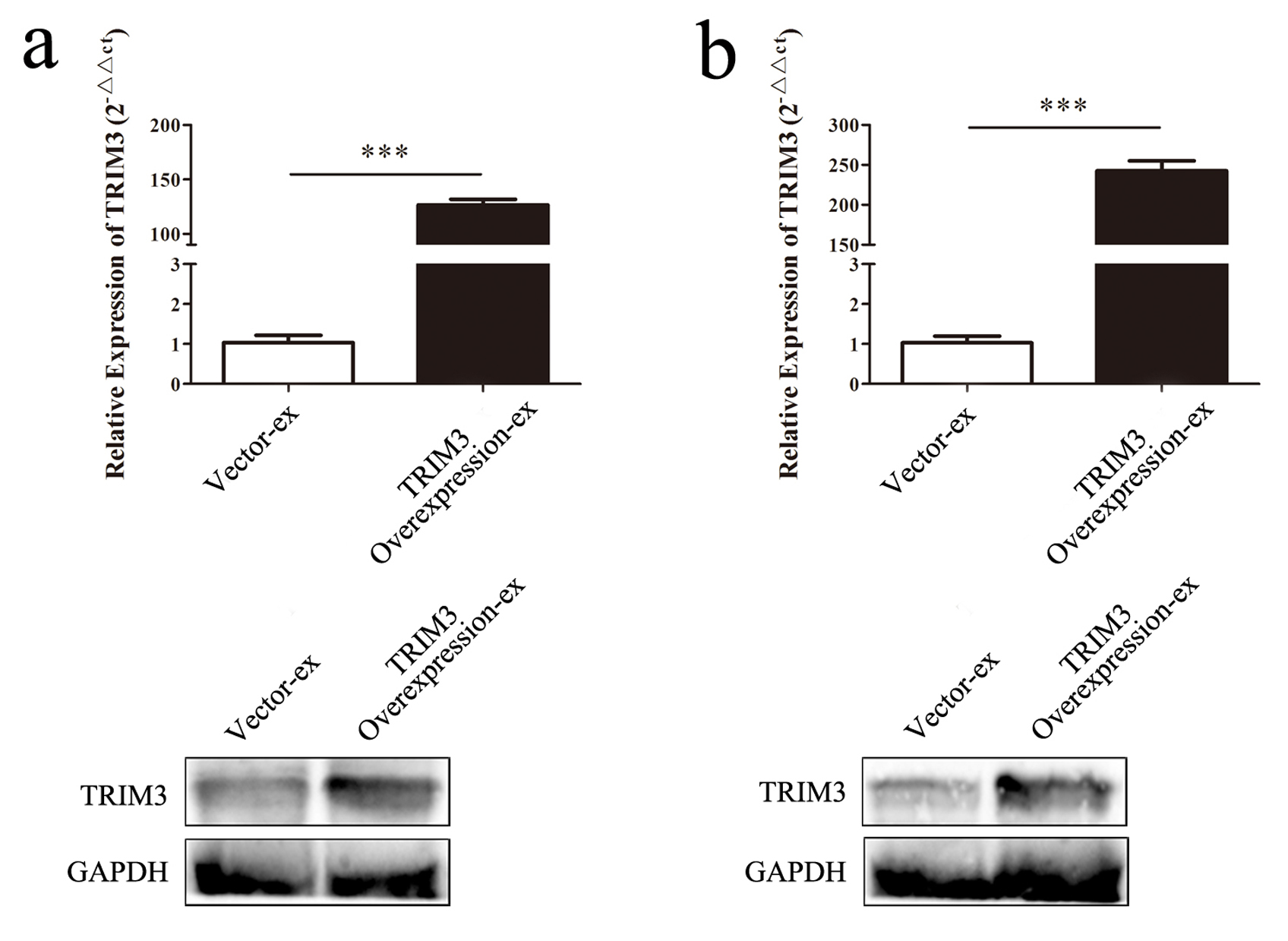

Supplement: Supplementary file 6 — Figure S3. The expression of TRIM3 in exosomes from gastric cancer cells transfected with TRIM3. a, MGC-803; b, SGC-7901; ***P < 0.001. (JPG 259 kb) [file 13046_2018_825_MOESM6_ESM.jpg]

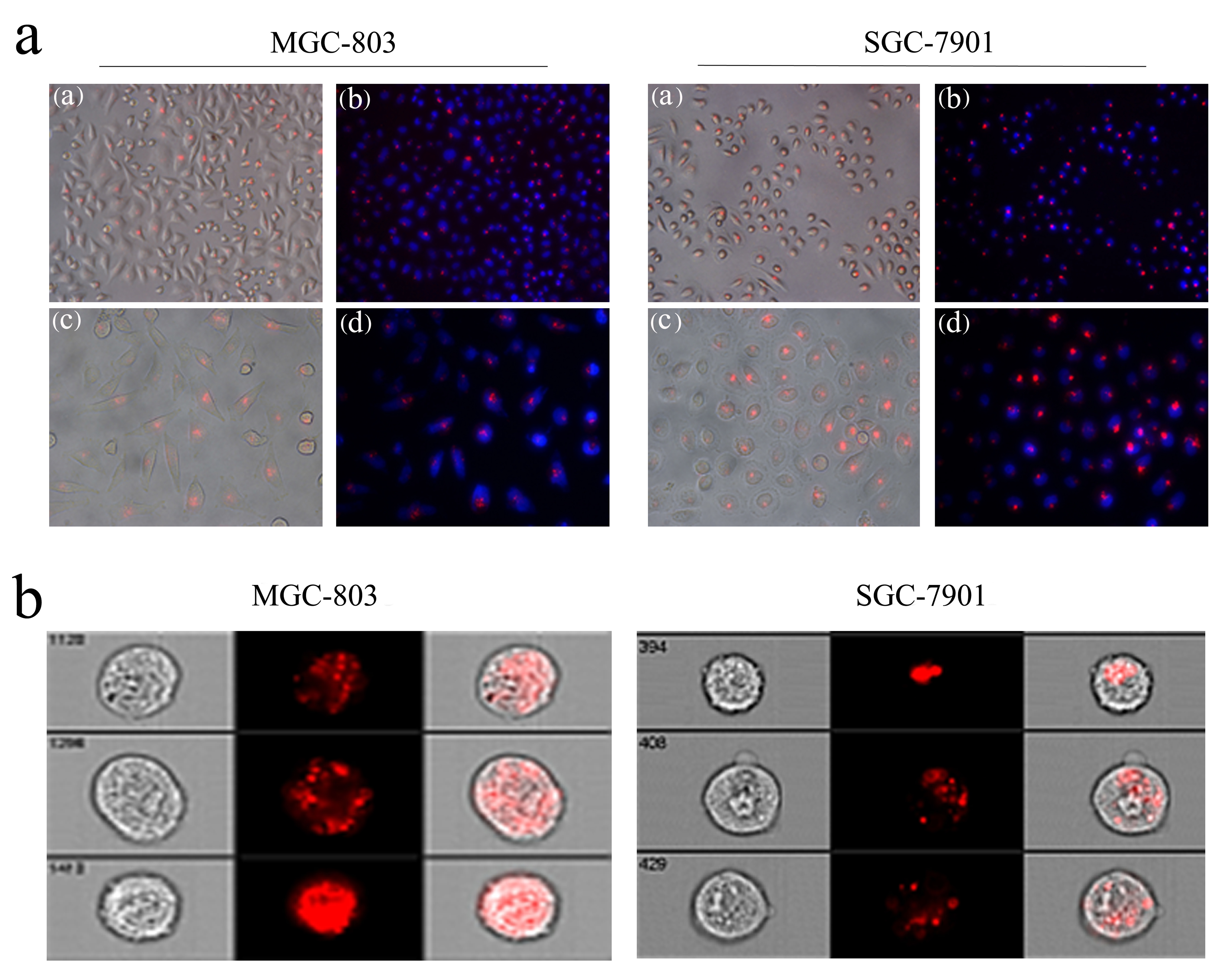

Supplement: Supplementary file 7 — Figure S4. Gastric cancer cells internalize exosomes. a, Exosome internalization into gastric cancer cells was determined by using confocal microscopy. Magnification, 200× ((a) and (b)); 400× ((c) and (d)). b, Exosome internalization into gastric cancer cells was determined by using imaging flow cytometry. (JPG 3089 kb) [file 13046_2018_825_MOESM7_ESM.jpg]

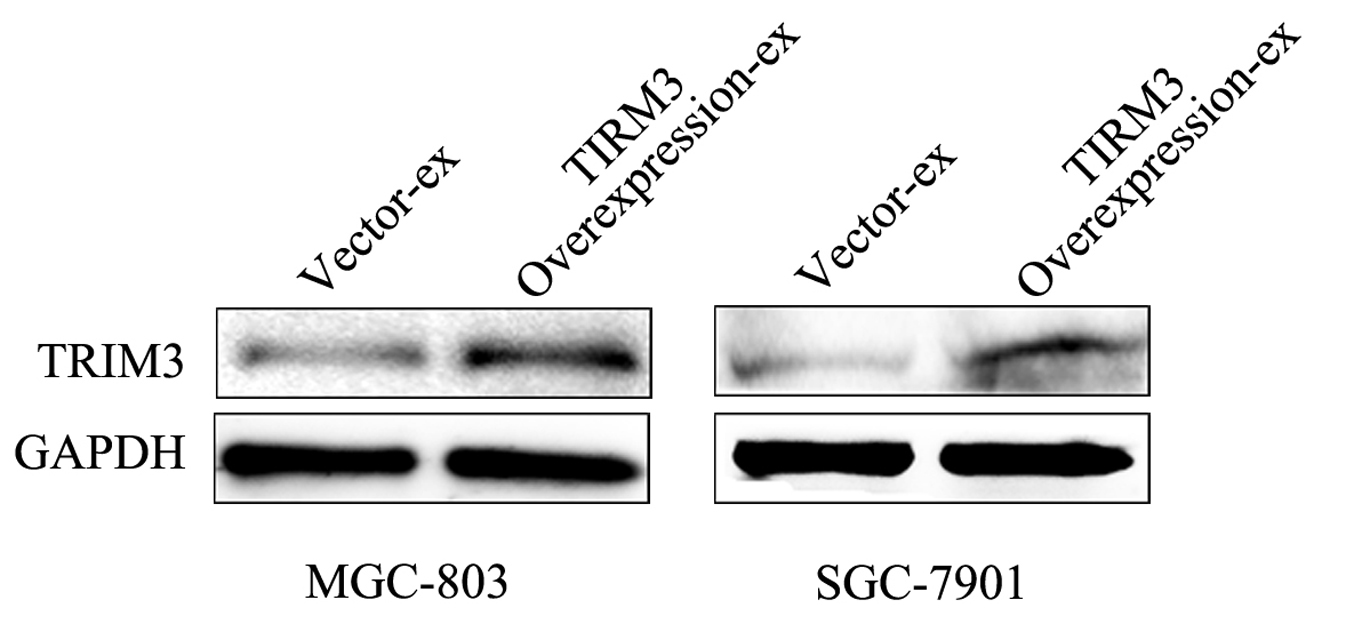

Supplement: Supplementary file 8 — Figure S5. The expression of TRIM3 in gastric cancer cells treated with control exosomes and TRIM3-overexpressing exosomes. (JPG 130 kb) [file 13046_2018_825_MOESM8_ESM.jpg]

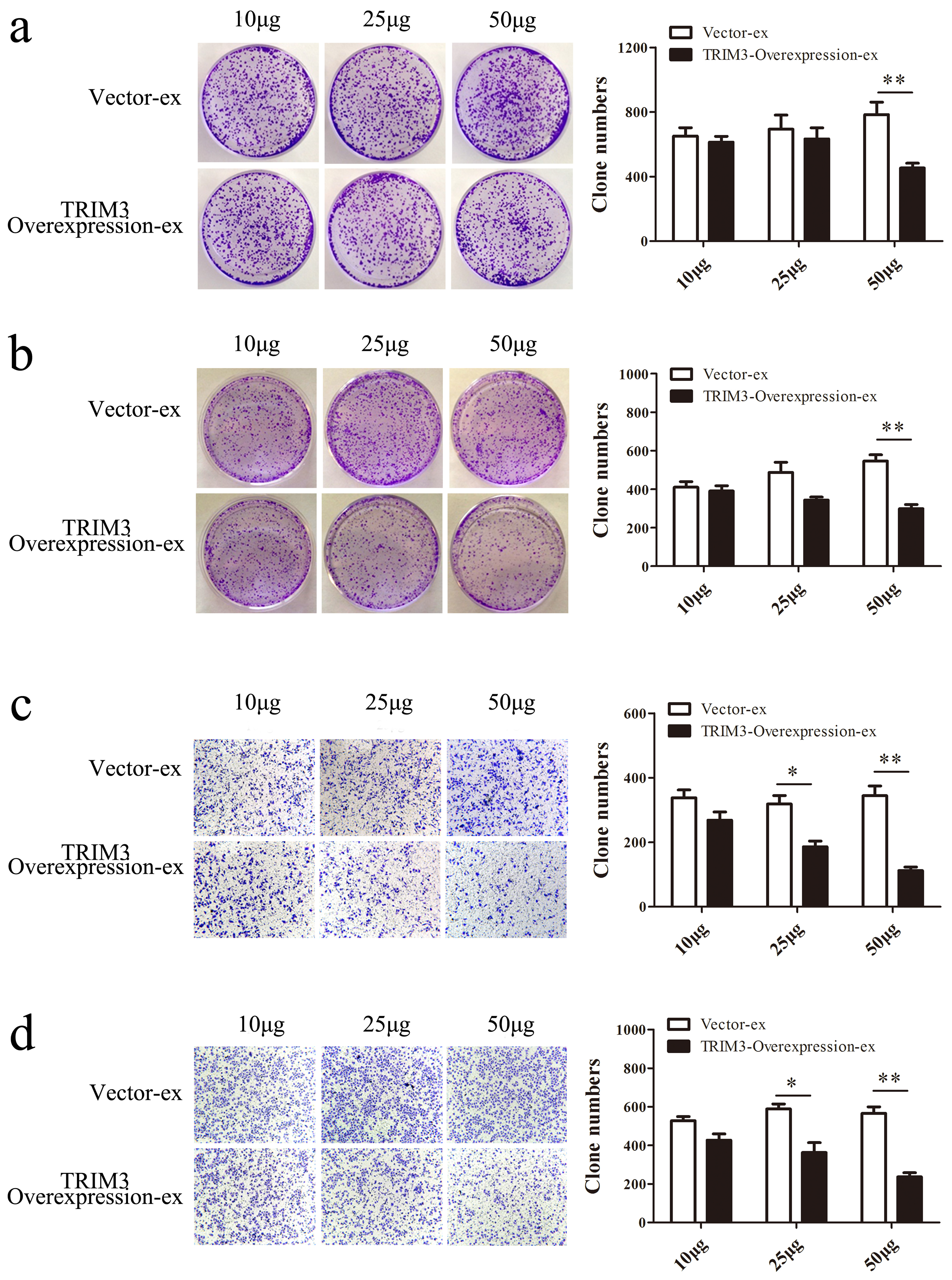

Supplement: Supplementary file 9 — Figure S6. The effects of TRIM3-overexpressing exosomes on the proliferation and migration of gastric cancer cells in vitro. a and b, The effects of TRIM3-overexpressing exosomes on the colony formation abilities of MGC-803 (a) and SGC-7901 (b) cells. **P < 0.01. c and d, The effects of TRIM3-overexpressing exosomes on the migration of MGC-803 (c) and SGC-7901 (d) cells. *P < 0.05, **P < 0.01. (JPG 5700 kb) [file 13046_2018_825_MOESM9_ESM.jpg]
